# *Apteranthes tuberculata*'s Antidiabetic Potential: Exploring Phytochemicals, Screening Antioxidant Activity, and Validating DPP‐4 Inhibition Using In Vitro and In Silico Approaches

**DOI:** 10.1002/fsn3.70494

**Published:** 2025-07-24

**Authors:** Ilham Khan, Shabana Bibi, Junaid Shehzad, Zarqa Riaz, Muhammad Saad Khan, Mansour Ghorbanpour, Murtaza Hasan, Ghazala Mustafa

**Affiliations:** ^1^ Department of Plant Sciences, Faculty of Biological Sciences Quaid‐i‐Azam University Islamabad Pakistan; ^2^ Department of Biosciences Shifa Tameer‐e‐Millat University Islamabad Pakistan; ^3^ Department of Biosciences Faculty of Sciences, COMSATS University Islamabad Sahiwal Pakistan; ^4^ Department of Medicinal Plants Faculty of Agriculture and Natural Resources, Arak University Arak Iran; ^5^ Department of Biotechnology The Islamia University of Bahawalpur Bahawalpur Pakistan; ^6^ State Agricultural Ministry Laboratory of Horticultural Crop Growth and Development, Ministry of Agriculture, Department of Horticulture Zhejiang University Hangzhou China

**Keywords:** ADMET, diabetes mellitus, DPP‐4 protein, glycosides, molecular docking

## Abstract

Diabetes is a chronic metabolic disorder that affects an increasing number of people worldwide, frequently managed with synthetic drugs that have side effects and can be costly. *Apteranthes tuberculata* (N.E.Br.) Meve & Liede, a plant with traditional medicinal use in Pakistan to treat diabetes, but its antidiabetic potential has not been scientifically validated. This research assessed the phytochemicals, antioxidant properties, and dipeptidyl peptidase‐4 (DPP‐4) inhibitory activity of *A. tuberculata* 's methanolic extract. The extract was assessed through in vitro antioxidant assays, DPP‐4 inhibition test, and metabolomic analysis via Fourier‐transform infrared (FTIR) spectroscopy and liquid chromatography–mass spectrometry (LC–MS). The study used computational tools to visualize compound structures, protein‐ligand interactions, and to measure pharmacokinetic parameters. Phytochemical analysis revealed significant levels of total phenols (71.991 ± 0.78 mg/g gallic acid equivalents) and flavonoids (66.216 ± 0.09 mg/g quercetin equivalents). Results showed a robust total antioxidant capacity (70.900 ± 2 mg/g ascorbic acid), total reducing power (72.000 ± 2.00 mg/g gallic acid equivalents), and DPPH IC_50_ value of 96.54 μg/mL. FTIR spectra showed the presence of carbohydrates and glycosides. The extract exhibited 70% DPP‐4 inhibitory activity (IC_50_ value = 46.761 ± 0.043 μg/mL), comparable to Sitagliptin at 78% (IC_50_ value = 20.474 ± 0.407 μg/mL). LC–MS identified 24 bioactive compounds, including flavonoids and glycosides, with compounds like Kaempferol‐3‐O‐rutinoside‐7‐O‐glucoside and Kaempferol‐7‐O‐rutinoside showing strong binding interactions with DPP‐4. These results underscore the therapeutic potential of *A. tuberculata* as a natural source of DPP‐4 inhibitors for managing diabetes.

## Graphical abstract

Metabolomics revealed 24 major compounds, mainly glycosides, flavonoids, and triterpenes. Antidiabetic compounds Kaempferol‐7‐O‐rutinoside and Kaempferol‐3‐O‐rutinoside‐7‐O‐glucoside show maximum inhibiting activity for DPP4 protein (in vitro and *in silico*). Out of 24 compounds, 5 demonstrate no PAINS and break alerts.

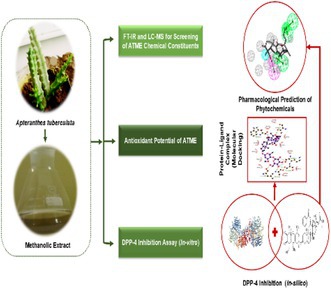

## 1 Introduction

*Apteranthes tuberculata*, belonging to the *Apocynaceae* family, is a genus with up to 200 species (Al‐Fatimi 2019). In Pakistan, two wild species, *A. tuberculata* and *A. edulis* , are highly drought‐resistant, leafless, and succulent herbs, typically found growing under the canopy of *Dodonaea viscosa* on exposed bared rocks (Abdel‐Sattar et al. 2007). The methanolic extract of *A. tuberculata* has been shown to possess antidiabetic, anti‐inflammatory, phytotoxic, antioxidant, and cytotoxic potential, as well as a shielding effect against gastric mucosa injuries and hematoprotective properties (Poodineh and Nakhaee 2016; Saif et al. 2021). Extracts of *A. tuberculata* have also been demonstrated to have antidiabetic activity in alloxan‐fed diabetic rabbits (Sultan et al. 2014), possibly through activation of insulin produced by pancreatic cells or extra‐pancreatic activation of peripheral glucose usage, as well as lipid‐lowering effects (Abdel‐Sattar et al. 2011). *A. tuberculata* has been demonstrated to improve antioxidant enzyme activity, regulate oxidative markers, and inhibit oxidative stress in diabetic patients (Poodineh et al. 2015). This medicinal plant is composed of a variety of compounds, including flavone glycosides, pregnane glycosides, megastigmane glycosides, saponins, bitter principles, and triterpenes (Abdel‐Sattar et al. 2013; Manzoor et al. 2022; Qasim et al. 2020). Gas chromatography (GC) analysis of *A. tuberculata* has further identified unstable compounds such as hydrocarbons, essential oils, and other fatty acids (Formisano et al. 2009; Zito et al. 2010). Metabolites, which are the end products of cell regulatory processes in response to environmental or genetic changes, are compiled in an organism's metabolome (Arshad and Mustafa 2023; Mustafa and Komatsu 2017, 2021). Metabolomics is a growing field that utilizes high throughput global investigation of targeted or untargeted metabolites to study their diverse functions in phytomedicines, toxicology, drug discovery, and development (Hasan et al. 2014; Zafar et al. 2024; Zaheer et al. 2023).

Plant metabolomes are thought to be more complex than those of animal cells, with an estimated number of metabolites exceeding 200,000. Each metabolite has a specific bioactivity corresponding to its structure (Sadaf et al. 2023; Shehzad et al. 2023). Recent interest in nutraceutical and pharmaceutical research has been sparked by the significant potential of plant secondary metabolites for drug discovery. While many such metabolites have been studied, very few novel drugs have been developed (Elshafie et al. 2023). Furthermore, plant metabolomics links phyto‐compounds with their presumed biological activities, yielding novel and important insights into medicinal plants. The experimental results of *A. tuberculata* strongly suggest that the plant contains a significant amount of active metabolic compounds, mainly glycosides, which can be used in the formulation of anti‐diabetic drugs (Hasan, Mehmood, et al. 2021; Ozturk et al. 2018). Diabetes mellitus (DM) is a complex metabolic disorder associated with various complications that reduce body activities due to serious damage to various body organs (Antar et al. 2023). Type 2 DM patients are often subject to serious complications such as cardiovascular disorders, renal disorders, blindness, amputation, myocardial infarction, and stroke (Lim and Park 2013). Various drugs are available for the treatment of DM, but no single drug can provide satisfactory results for all conditions of DM patients (Centers for Disease Control and Prevention 2014).

DPP‐4 inhibitors are among the important therapeutic classes of DM drugs, and several such inhibitors, such as sitagliptin, saxagliptin, linagliptin, and alogliptin, have been approved by the FDA and are available on the market (Pawaskar et al. 2019; Wu et al. 2016). Yet presently available synthetic DPP‐4 inhibitors are frequently linked with undesirable side effects, high costs, and limited long‐term medication impact. These challenges have encouraged interest in the search for budget‐friendly, safer, and more efficient plant‐based alternatives.

*Apteranthes tuberculata* has been locally used in traditional medicine but remains mainly underexplored in scientific literature. Even though research has examined certain phytochemicals in this plant, no thorough investigation exists about its antidiabetic properties through DPP‐4 inhibition. This research signifies the first thorough report from Pakistan assessing the in vitro DPP‐4 inhibitory potential of *A. tuberculata* , supplemented by untargeted metabolomic profiling and computational validation, promoting its novelty and scientific contribution. Addressing a significant gap in current literature, as no prior studies have reported DPP‐4 inhibition of this plant. Metabolomics, specifically untargeted using LC–MS and FTIR approaches, enables effective detection and identification of various bioactive metabolites found in medicinal plants. The methods reveal compounds that could affect DPP‐4 inhibition as well as correlation with biological activity. This research integrates metabolomics with in vitro and *in silico* analysis to discover *A. tuberculata* compounds with lead potential for plant‐based antidiabetic drug development. Taken together, the novelty of this study lies in being the first to report the DPP‐4 inhibitory activity of *A. tuberculata* using omics and pharmacological approaches, filling a key gap in the literature about its therapeutic capability and supplementing its significance as a candidate for future drug discovery.

## 2 Materials and Methods

The present experiment was carried out in the Plant Proteomics laboratory at Quaid I Azam University Islamabad. The study was used to analyze the phytochemical properties, antioxidant potential, and metabolomic profiling of *A. tuberculata* . Fresh plant samples were collected from the Lower Dir district of Khyber Pakhtunkhwa (34°51′ N 71°51′ E). The plant specimen was taxonomically identified by Associate Prof. Dr. Muhammad Zafar. The botanical nomenclature was further verified using the WFO database (https://wfoplantlist.org/taxon/wfo‐0000540983‐2024‐12?page=1).

### 2.1 Chemicals Used

Folin–Ciocalteu Reagent (Sigma‐Aldrich, US), Na_2_CO_3_ (Sigma‐Aldrich, US), Gallic Acid (Sigma‐Aldrich, US), DPPH (Sigma‐Aldrich, US), Ascorbic Acid (Sigma‐Aldrich, US), Potassium Ferricyanide (Sigma‐Aldrich, US), Gallic Acid (Sigma‐Aldrich, US), Ammonium Molybdate (Sigma‐Aldrich, US), Gly‐Pro‐Pnitroanilide (Sigma‐Aldrich, US), DPP‐4 (Sigma‐Aldrich, US), Sitagliptin (Sigma‐Aldrich, US), DMSO (Sigma‐Aldrich, US), Methanol (Sigma‐Aldrich, US), TCA (Sigma‐Aldrich, US).

### 2.2 Extract Preparation

The Arial part of the plant was washed with tap water to remove soil particles and other impurities and shade dried. After the complete drying, the plant material was ground into fine powder in grinder (Silver Crest Powder Grinder Machine Model SC‐ 150G). The ground plant material of 15 g was dissolved in 150 mL methanol in a 250 mL Erlenmeyer flask. The flask was then kept in a shaker for 48 consecutive hours at room temperature. After that extract was filtered through Whatman No. 1 filter paper. The filtrate was evaporated using a rotary evaporator (Rotavapor R‐300, BUCHI Labortechnik GmbH, Switzerland) and further dried in a vacuum oven (Shehzadi et al. 2022). The process was repeated twice. The resulting crude extract was stored at 4°C for further use. The extraction yield was calculated to be 26.67%.

### 2.3 Qualitative Phytochemical Analysis

Methanolic extract of *A. tuberculata* was tested for the presence or absence of bioactive secondary metabolites using a method previously reported by (Jabeen et al. 2023).

### 2.4 Quantitative Phytochemical Analysis

#### 2.4.1 Total Phenolic Contents

The total phenolic contents TPC in plant extract were determined with a Folin–Ciocalteu assay. Aliquots of 20 μL of the extract were added to 90 μL of Folin–Ciocalteu reagent in a 96‐well plate. Then, 20% of aqueous Na_2_CO_3_ solution (90 μL) was added and mixed well. Absorbance was recorded at 760 nm after 2 h. Using gallic acid as a standard, a calibration curve was prepared with various concentrations (15.625–500 μg/mL). The total phenolic contents were expressed as mg gallic acid equivalent (Farag et al. 2020; Hasan et al. 2020; Hasan, Gulzar, et al. 2021).

#### 2.4.2 Total Flavonoid Contents

Total flavonoid contents in plant extract were estimated using (Farag et al. 2020) method. Aliquots of 20 μL of the extract were added to 10 μL of a 2% ethanolic solution of AlCl_3_ in a 96‐well plate. Distilled water was added to make the mixture 200 μL. Absorbance was determined at 415 nm after incubation for 30 min at 37°C. Using quercetin as a standard collaboration curve was prepared with various concentrations (3.125–100 μg/mL). The amount of total flavonoid content was calculated from the quercetin calibration curve, and results were expressed as quercetin mg/g equivalent.

### 2.5 Radical Scavenging Assay

The antioxidant activity of *A. tuberculata* extract was estimated with the DPPH in vitro method with some modifications. An aliquot of 10 μL was mixed with a 0.004% methanolic solution of DPPH (190 μL). The mixture was left to stand for 30 min in the dark. Then, the absorbance was recorded at 517 nm. Ascorbic acid was used as a positive control. Serial dilutions of ascorbic acid were prepared at different concentrations (62.5–4000 μg/mL).

DPPH radical scavenging activity (RSA) in percentage was calculated by the following equation.

$$\mathrm{RSA}\%=\left[\;\left(A_{0}–A_{S}\right)/A_{0}\right]\times 100$$

A_0_ = control absorbance.

A_S_ = sample absorbance at 517 nm (Farag et al. 2020).

### 2.6 Total Reducing Power

Reducing power of *A. tuberculata* methanolic extract was carried out according to the procedure (Farag et al. 2020) of with slight modifications. Various concentrations of methanolic extract (62.5–4000 μg) were dissolved in 1 mL of methanol with potassium ferricyanide (250 μL, 1%) and phosphate buffer (200 μL, 0.2 mol/L, and pH 6.6). Then 200 μL of 10% TCA (trichloroacetic acid) was added after incubation for 20 min at 50°C. The mixture was centrifuged at 3000 rpm for 10 min at room temperature. Then 50 μL of distilled water was added to the upper layer (150 μL) and ferric chloride (50 μL, 0.1%). Absorbance was recorded at 700 nm. Results were expressed as the equivalent of mg gallic acid (GAE/mg) equivalent.

### 2.7 Total Antioxidant Capacity

To determine total antioxidant activity, the phospho‐molybdenum method was followed. Plant extract 100 μL was added to 900 μL reaction solution (28 mM sodium phosphate, 0.6 M H_2_SO_4_, and ammonium molybdate 4 mM). Then the mixture was incubated for 90 min at 95°C. The mixture was then allowed to cool at room temperature and absorbance was determined at 695 nm. Results were expressed as ascorbic acid equivalent.

### 2.8 Fourier Transform Infrared Spectroscopy (FTIR)

Fourier transform infrared spectroscopy is the most influential tool used to detect the functional groups or bonds present in plant compounds. The relevant form of the bond can be seen and interpreted from the spectrum when light is absorbed at a specific wavelength. The chemical bonds or functional groups present in a compound can be identified by deciphering the absorption spectrum (Nagarajan and Kumar 2017). The methanolic extract of plant 100 mg was loaded in an FTIR spectroscope (DW‐FTIR‐530A, China) for analysis. The absorbance of the sample was recorded on the wavelength range from 4000 to 400 cm^−1^.

### 2.9 Liquid Chromatography‐Mass Spectrometry (LC–MS)

The fresh plant sample was taken, cut into pieces, and frozen in liquid nitrogen. 500 mg of frozen tissue was ground to a fine powder in a precooled pestle and mortar in liquid nitrogen. The powdered plant material was transferred to a 50 mL falcon tube (precooled). After that, 8 mL of chilled extraction solution was added. The mixture was vortexed for 10 s and sonicated for 15 min at room temperature. Then the centrifugation of the sample was done at 3000 rpm at room temperature. The supernatant was filtered through a 0.2‐μm syringe filter in a 2 mL glass vial and was closed with a cap. Then the sample was analyzed using LC–MS (LCMS‐9050 Shimadzu, Japan) (Iqbal et al. 2013, 2014; De Vos et al. 2007).

### 2.10 In Vitro DPP‐4 Inhibition Assay

The assay was conducted as previously described by (Quek et al. 2021), with several modifications. The experiment was carried out in triplicate in a 96‐well microplate with a total volume of 200 μL. The plant extract was dissolved in DMSO (Dimethyl Sulfoxide) at concentrations of 7.8125, 15.625, 31.25, 62.5, 125, 250, 500, 1000, 2000, and 4000 mg/mL. A mixture of 45 μL Tris–HCl buffer, 40 μL of plant extract, and 15 μL of DPP‐4 enzyme solution (0.05 U/mL) was preincubated for 10 min at 37°C, after which 50 μL of Gly‐pro‐pnitroanilide (GPPN 0.2 mM in Tris–HCl) was added to the mixture. The final incubation was done at 37°C for 30 min, and the absorbance was measured at 405 nm using a plate reader. Sitagliptin was used as a positive control. Percent enzyme inhibition was calculated using the following formula:

$$\text{Percent inhibition}=\left[\;\left(\text{A0}–\mathrm{AS}\right)/\text{A0}\right]\times 100$$

A0 = control absorbance.

AS = sample absorbance.

### 2.11 Molecular Docking

Experimentally identified 21 compounds were used as a dataset for molecular docking studies. Their two‐dimensional structure was drawn by ChemDraw Ultra 12.0 (Table S3) (Cousins 2011). The three‐dimensional structure of dipeptidyl peptidase‐IV (DPP‐IV) protein was retrieved from a protein data bank with accession ID 5I7U with a resolution 1.95 Å (Figure 5) (Wu et al. 2016). AutoDock Vina was used for the molecular docking of selected 21 compounds (Trott and Olson 2010). The initial step was followed by the preparation of the target protein and ligand structure for the calculation of physiochemical properties and converted from CDX (ChemDraw Exchange) files to mol format and then converted into PDB format by Open Bebel (O'Boyle et al. 2011). Ligand and protein structure files in PDB format were subjected to the AutoDock module for the generation of PDBQT files with the addition of charges and missing hydrogen atoms The grid set points were well‐defined for MD, and after running the script, the top ten docked complex models were generated, with the lowest energy model selected based on the best ligand pose in the binding pocket.

### 2.12 Protein‐Ligand Interaction Analysis

The selected best models with significant energy values were imported to Ligplot+ software (Laskowski and Swindells 2011) for the investigation of protein‐ligand binding interactions. Their two‐dimensional plots were generated, demonstrating the hydrogen and hydrophobic interactions within the distance range of 4 Å and the three‐dimensional confirmations (Figures 6 and 7). The docked complex was visualized in chimera software showing the binding pocket of the target protein and the hydrophobic nature of the DDP‐4 target protein (Pettersen et al. 2004).

### 2.13 Pharmacological Prediction

In computer‐aided drug design protocols, *in silico* ADMET (absorption, distribution, metabolism, excretion, and toxicity) profile estimations are acceptable, so ADMET properties were calculated by SwissADME (Daina et al. 2017) and DataWarrior (Sander et al. 2015) tools are shown in Table 2.

### 2.14 Statistical Analysis

Statistical analysis was conducted using SPSS software (version 27; IBM Corp., Armonk, NY, USA). Data were expressed as mean ± standard deviation (SD) from triplicate assays (*n* = 3). One‐way analysis of variance (ANOVA) followed by Tukey's post hoc test was applied to determine statistically significant differences between group means at a significance level of *p* < 0.05.

## 3 Results and Discussion

### 3.1 Qualitative and Quantitative Analysis

Qualitative phytochemical analysis of *A. tuberculata* methanolic extract is given in (Table S1). The result indicates that flavonoids and phytosterols are present in higher amounts followed by saponins and tannins. Quantitative phytochemicals were analyzed such as phenolics and flavonoids. In our study, total phenolics and flavonoid content present in CTME were (71.991 ± 0.78 mg/g GAE) and (66.2162 ± 0.09) respectively (Table S2). (Maheshu et al. 2014) determined the total phenolic content of *C. adscendens* methanolic extract was (21.0 ± 0.59 mg/g GAE) and flavonoid content was (3.7 ± 1.2 mg/g QE). According to Chandran phenolic and flavonoid contents of *C. diffusa* were (6.26 g/100 g GAE) and (8.7 g/100 g RE) respectively (Chandran et al. 2014). When compared to the previous study the phenolic and flavonoid contents of *A. tuberculata* are expressively higher than other species of *Caralluma*. The flavonoid and phenolic contents observed in the current work might be helpful in free radical scavenging or antioxidant activity. The quantity of phenolics, specifically flavonoids content existing in the methanolic extract of the plant may evidence the antioxidant activity of the plant. Hence the occurrence of flavonoids and phenolics content supports that *A. tuberculata* can have antioxidant potential.

### 3.2 Antioxidant Activity

**FIGURE 1:**
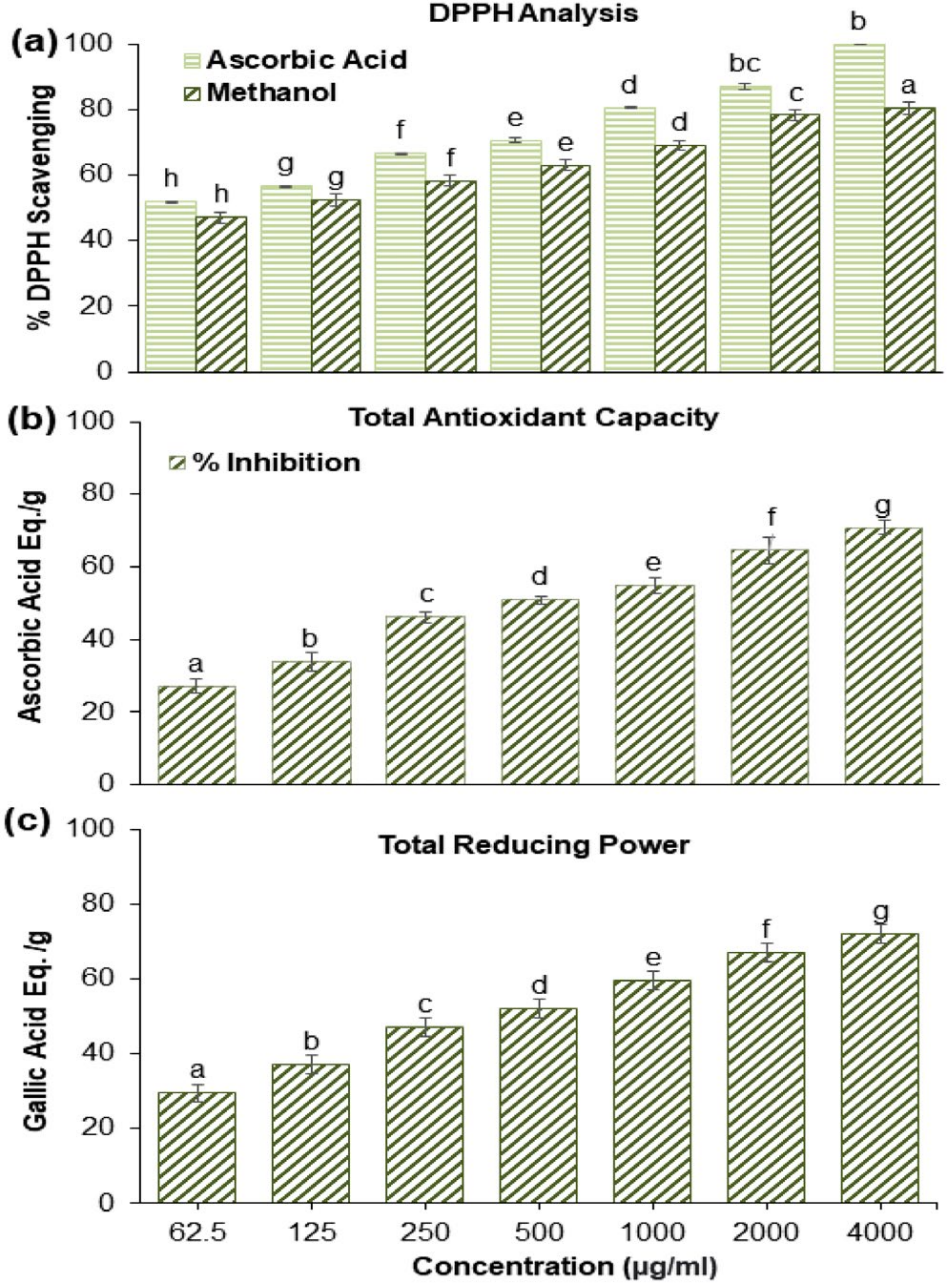
(a) Shows the percentage inhibition of DPPH radical scavenging activity by ATME at different concentrations. (b) Demonstrate the total reducing power of the ATME at various concentrations. (c) Represents the antioxidant capacity of ATME. Antioxidant activities increase with higher concentrations, indicating a dose‐dependent relationship. Concentrations for all activities range from 62.5 to 4000 μg/mL. The results are presented as the mean ± S.D. of three independent biological replicates. Mean values at each point that differ from one another are denoted by different letters and are considered significantly different according to one‐way ANOVA (*p* < 0.05). ATME, *Apteranthes tuberculata* methanolic extract.

In the current study, the result shows that radical scavenging activity is dose dependent. The calculated IC_50_ value from the percentage inhibition of DPPH was 96.54 μg/mL for ATME. Figure 1a shows the percentage inhibition of ATME and ascorbic acid at different concentrations. Similarly, Figure 1b shows the reductive competence of ATME equated to gallic acid (GA). In the present study, the highest reducing power was observed at 4000 μg/mL, which was (72.000 ± 2.00 mg/g of gallic acid) and the lowest value was observed at a concentration 62.25 (29.625 ± 2.315 mg/g of GAE). The result shows that an increase in concentration increases the reducing capacity of the plant extract. Like total reducing capacity, total antioxidant capacity (TAC) also increased as the plant sample increased (Figure 1c). The present study reveals that maximum TAC was observed at a concentration of 4000 μg/mL, which was (70.900 ± 2 equivalent to ascorbic acid mg/g of extract) and lowest at 62.5 (27.233 ± 2.08 AAE mg/g of extract). The radical scavenging activity increased with an increase in concentration and the IC_50_ value recorded was 96.54 μg/mL for the methanolic extract of the plant sample. Our results are according to (Rauf et al. 2013), who also reported that the radical scavenging assay is dose dependent. According to (Al‐Fatimi 2019), the IC_50_ value of *C. adscendens* methanolic extract was 66 μg/mL. Maximum antioxidant activity and total reducing power were observed at 4000 μg/mL, which is (70.900 ± 2 mg/g AA) and (72.000 ± 2.00 mg/g GAE) respectively, which is also reported by (Chandran et al. 2014) in *C. diffusa* .

### 3.3 Fourier Transform Infrared Spectroscopy (FTIR)

**FIGURE 2:**
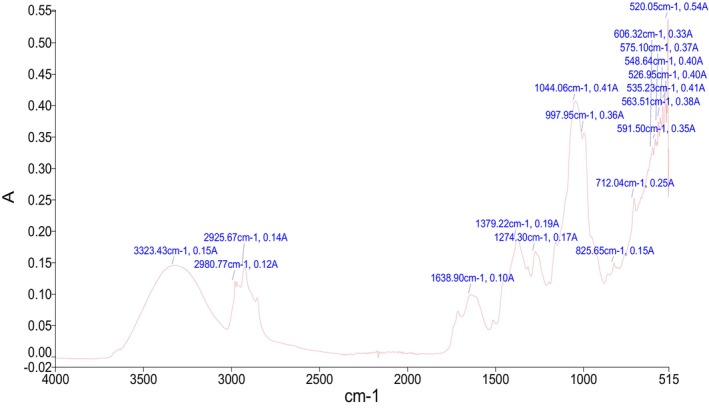
FT‐IR spectra display the functional group of inorganic and organic compounds in *A. tuberculata* extract. The spectra demonstrate characteristic absorption peaks analogous to different functional groups identified in the plant extract. These functional groups are linked to the plant's therapeutic properties and bioactive potential, including antidiabetic and antioxidant activities.

In the current study, functional groups were examined using FTIR spectroscopy based on their absorbance. Figure 2 shows the characteristic FTIR spectra of *A. tuberculata* . The peaks obtained from the analysis were decoded, and the functional groups were determined. The functional groups present in the plant sample refer to a specific class of compounds (Table S4). The frequently occurring compounds were carbohydrates and glycogen, which refer to the presence of glycosides, a class of bioactive phytochemicals known for their antidiabetic potential. The valuable health‐enhancing properties of *A. tuberculata* are due to the presence of bioactive compounds. The FTIR reveals that the major compounds in the plant sample are carbohydrates. Frequencies range from 3323 cm^−1^, 2980 cm^−1^, and 2925 cm^−1^ characterizing O‐H stretching and the existence of carbohydrates and amino acids. Carbohydrates and glycosides are known to demonstrate glucose‐lowering and antioxidant effects, aiding in the therapeutic properties of the plant (Khattak and Khan 2016). Frequency ranges from 1638 cm^−1^ characterize N‐H bend and the existence of proteins, which may play a role in modifying enzymatic activities related to diabetes. Frequencies range from 1379 cm^−1^ exhibit C‐H bend, indicating the presence of glycogen. Frequencies range from 1274 cm^−1^ and 1044 cm^−1^ represent C‐N stretching and the presence of amino acids. Frequencies range from 997 cm^−1^ represent = C‐H bend, indicating the presence of lipids, which are crucial for cell membrane integrity and may also induce insulin sensitivity (Rizevsky et al. 2022). Frequencies range from 825 cm^−1^ and 712 cm^−1^ represent C‐H stretching and the presence of phenolics, extensively identified for their robust antioxidant potential (Johnson et al. 2020). The frequency ranges from 606 cm^−1^, 591 cm^−1^, 575 cm^−1^, and 563 cm^−1^ represent C‐Cl stretching and the existence of chloro‐compounds as previously reported and matched (Hasan et al. 2024; Mustafa et al. 2020, 2024; Shehzad et al. 2024). Frequencies range from 548 cm^−1^, 535 cm^−1^, 526 cm^−1^, and 520 cm^−1^ represent C‐Br stretching and the existence of bromo‐compounds. Similar results were also reported by (Nagarajan and Kumar 2017) from garlic by analysis through FTIR.

### 3.4 Liquid Chromatography‐Mass Spectrometry (LC–MS)

**FIGURE 3:**
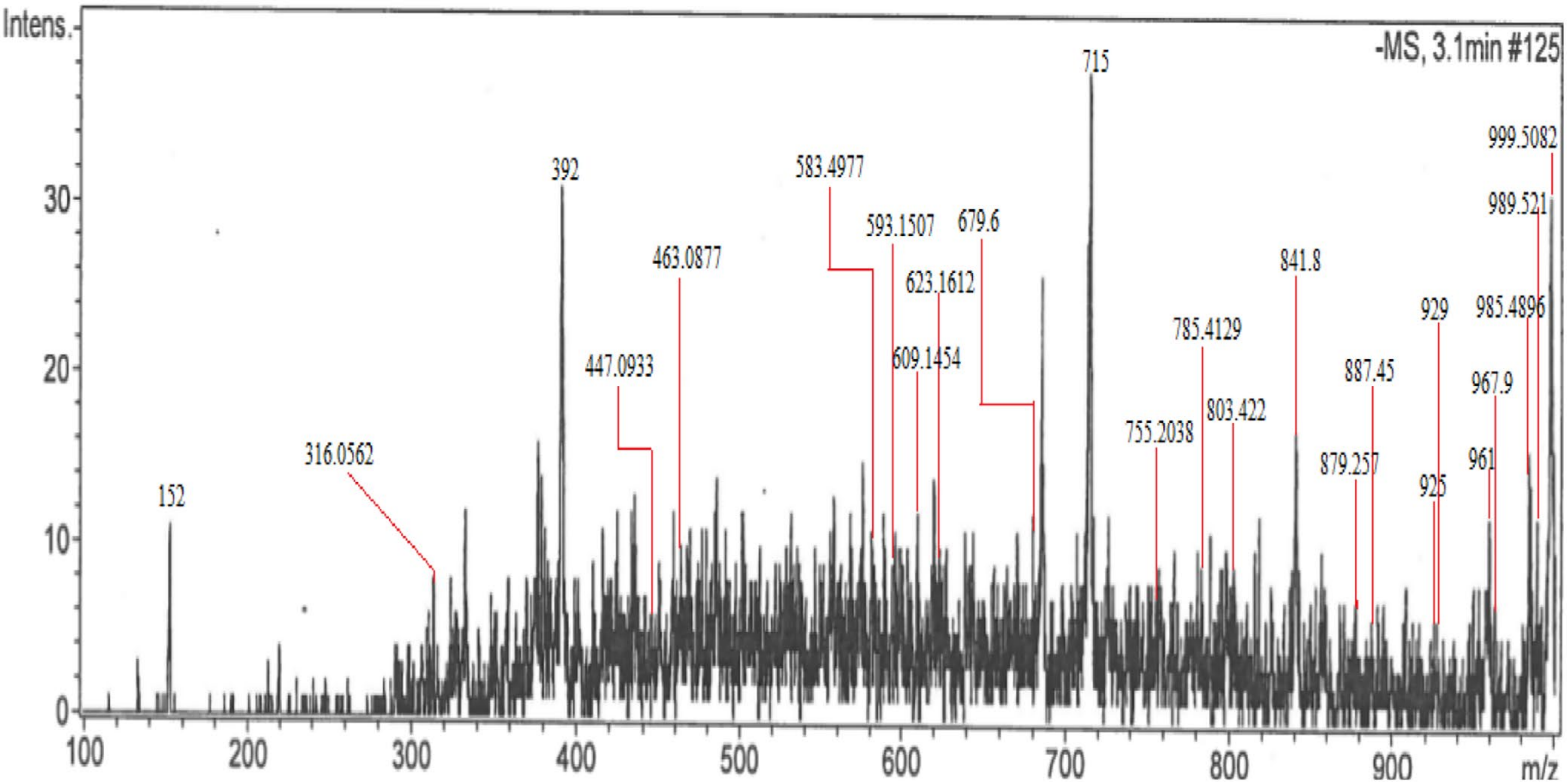
Representative LC–MS chromatogram of analyzed sample. LC–MS untargeted screening of bioactive secondary metabolites was carried out to evaluate the therapeutic potential of *A. tuberculata* plant.

In this study, we carried out an untargeted screening of bioactive secondary metabolites in *A. tuberculata* to evaluate its therapeutic potential. A total of 24 compounds were identified in Table 1 with retention time ranging from 1 to 20 min and mass‐to‐charge ratio (m/z) ranging from 152 to 1000 (Figure 3). Our result shows that acylated glycosides are the most abundant class of compounds, frequently present in all species of *Caralluma*. *A. tuberculata* is traditionally used for diabetes due to the presence of these acylated glycosides. In addition, we identified other important classes of secondary metabolites such as flavonoids, saponins, and sesquiterpene lactones. The LC–MS study of the methanolic extract of *A. tuberculata* displayed a diverse range of molecular ions spanning an m/z ratio of 152 to 1000 within the plant extract. Previous studies have explained structures of various acylated pregnane glycosides (7, 11, 13, 15, 19, 20, 21) using techniques such as FAB‐MS, NMR, HMBC, and HMQC (Abdel‐Sattar et al. 2008). The presence of acylated or nonacylated pregnane glycosides, such as russelioside C, raratuberside C, and russelioside B, in *A. tuberculata* indicates its antidiabetic activity. This is due to the blocking of G‐6‐Pase activity, stimulation of insulin release, improvement of glucose consumption, and inhibition of glucose uptake.

**TABLE 1:** List of identified bioactive secondary metabolites through LC–MS in *A. tuberculata* .

S.No	t_R_/min	[M‐H]^−^ (m/z)	Formula	Identification
1	1.4	152	C_5_H_5_N_5_O^+^	Deoxyguanosine
2	4.8	316.0562	C_16_H_12_O_7_	Isorhamnetin
3	5.2	325.0926	C_15_H_17_O_8_	Bilobalide
4	7.4	423.1295	C_20_H_23_O_10_	Ginkgolide J
5	7.9	447.0933	C_21_H_19_O_11_	Kaempferol‐7‐*O*‐glucoside
6	8.2	463.0877	C_21_H_19_O_12_	Quercetin‐3‐*O*‐glucoside
7	10.8	583.4977	C_51_H_76_O_17_	Caratuberside E
8	11.21	593.1507	C_27_H_29_O_15_	Kaempferol‐7‐*O*‐rutinoside
9	11.63	609.1454	C_27_H_29_O_16_	Quercetin‐3‐*O*‐rutinoside
10	11.8	623.1612	C_28_H_31_O_16_	Isorhamnetin‐3‐*O*‐rutinoside
11	12.9	679.6	C_34_H_56_O_12_	Russelioside C
12	14.6	755.2038	C_33_H_39_O_20_	Kaempferol‐3‐*O*‐rutinoside‐7‐*O*‐glucoside
13	15.5	785.4129	C_43_H_62_O_14_	Russelioside G
14	15.8	803.422	C_14_H_64_O_14_	Digoxin
15	16.4	841.8	C_40_H_66_O_17_	Russelioside B
16	17.3	879.257	C_40_H_47_O_22_	Ginkgolide C
17	17.5	887.45	C_46_H_66_O_14_	Pregnane glycoside
18	18.3	925	C_49_H_74_O_15_	Bouceroside—ADC
19	18.4	929	C_50_H_47_O_17_	Russelioside F
20	19	961	C_40_H_66_H_17_	Caratuberside E
21	19.3	967.9	C_51_H_76_O_16_	Raratuberside C
22	19.5	985.4896	C_54_H_74_O_15_	Isopropenyl Glycopyranosides
23	19.6	989.521	C_51_H_76_O_16_	Tragopogonsaponin M
24	19.9	999.5082	C_55_H_76_O_15_	12,20‐di‐*O*‐benzoyl boucerin

Furthermore, in this study the identified compounds such as isopropenyl glycopyranosides and 12,20‐di‐*O*‐benzoyl boucerin, were previously reported by (Sultan et al. 2014) from chloroform extract of *C. quadrangular*. Using MS and NMR data, structures of compounds 17 and 22 were elucidated as 12‐β‐*O*‐benzoyl‐20‐*O*‐tigloyl boucerin‐3‐*O*‐β‐_D_‐glucopyranosyl‐(1 → 4)‐β‐_D_‐cymaropyranoside and 12,20‐di‐*O*‐benzoylboucerin 3‐*O*‐β‐_D_‐cymaropyranoside (Abdel‐Sattar et al. 2008). Moreover, we identified flavonoid glycosides (compounds 5, 6, 8, 9, 10, and 12), including mono‐ and di‐glycosides with basic structure. Although, these compounds have been previously reported by (Lim et al. 2023; Lim and Park 2013) (Zhou et al. 2014) from *Ginkgo biloba* but identified here for the first time in *A. tuberculata* . The structures of these compounds were elucidated through UHPLC‐LTC‐Orbitrap Elite (Ablajan and Tuoheti 2013). Compound 14 was identified as digoxin, a cardiac glycoside previously isolated from *Digitalis lanata* for the first time. Our results strongly indicate that *A. tuberculata* contains a significant amount of active metabolic compounds with medicinal properties, particularly for the treatment of diabetes and potentially other diseases.

### 3.5 In Vitro DPP‐4 Inhibition Assay

**FIGURE 4:**
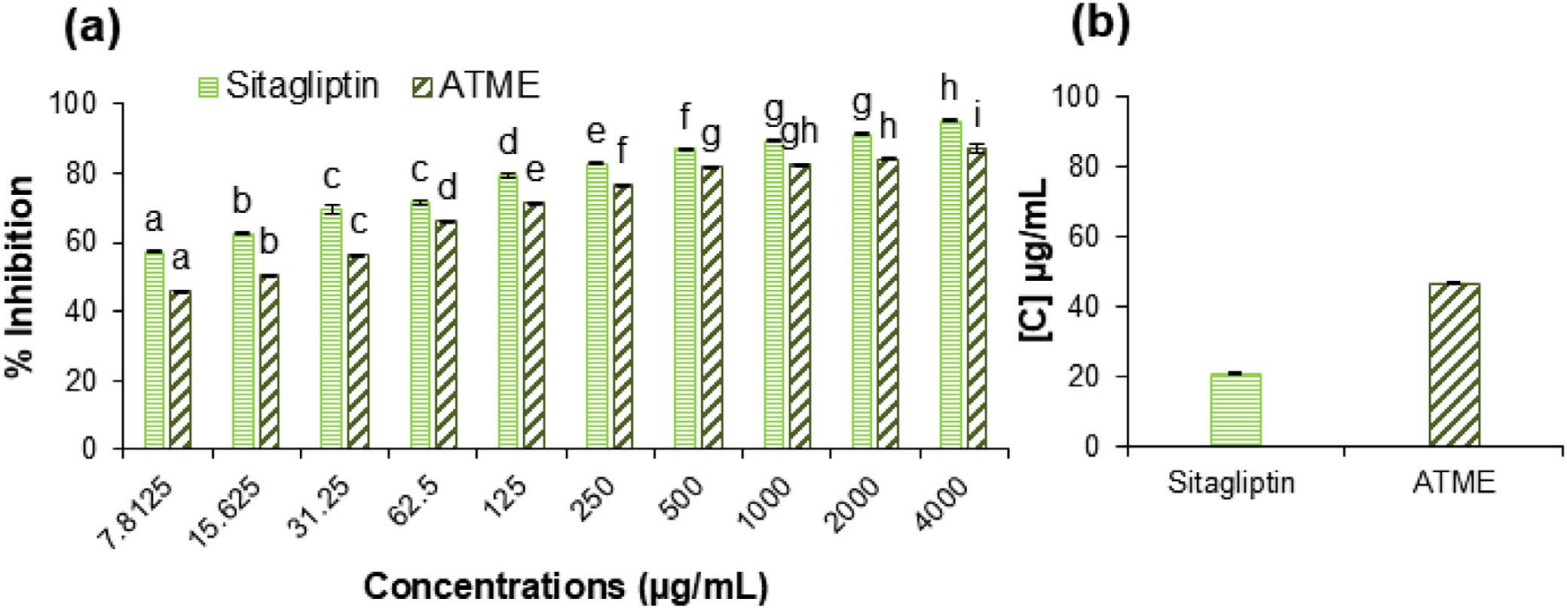
(a) In vitro DPP‐4 inhibition assay of ATME. Percentage inhibition was determined at different concentrations (7.8125–4000 μg/mL). Result shows that percentage inhibition is dose‐dependent, and the activity increased with an increase in concentration. (b) Calculated IC_50_ value of plant extract and sitagliptin (drug) from the percentage inhibition of DPP‐IV. The results are presented as the mean ± S.D. of three independent biological replicates. Mean values at each point that differ from one another are denoted by different letters and are considered significantly different according to one‐way ANOVA (*p* < 0.05). ATME; *Apteranthes tuberculata* methanolic extract.

DPP‐IV, or dipeptidyl peptidase‐IV, is an enzyme that breaks down certain peptides (chains of amino acids) in the body, including the incretin hormones GLP‐1 and GIP. These hormones are important for glucose metabolism and play a role in the body's response to insulin (Lok et al. 2022). Plant metabolites such as flavonoids, terpenoids, and alkaloids have been studied for their potential as DPP‐4 inhibitors. For example, flavonoids such as quercetin, kaempferol, and luteolin have shown inhibitory effects on DPP‐IV. Similarly, terpenoids such as glycyrrhizic acid and oleanolic acid are potent inhibitors of DPP‐4 (He et al. 2019). Alkaloids such as berberine and piperine have also been studied for their potential as DPP‐4 inhibitors. These plant metabolites play an important role in regulating glucose levels and thus possess therapeutic potential for the treatment of diabetes (Marahatha et al. 2021). The LC–MS result of *A. tuberculata* demonstrated high amounts of secondary metabolites such as flavonoids, terpenoids, and alkaloids. As a result, it showed a good DPP‐4 inhibitory activity with an IC_50_ value of 46.761 ± 0.043 μg/mL (70% inhibition) (Figure 4a,b). This is the first report of DPP‐4 inhibitory activity of *A. tuberculata* . To compare the effect, sitagliptin was used as a positive control and it showed an IC_50_ value of 20.474 ± 0.407 μg/mL (78% inhibition) (Figure 4a,b). The secondary metabolites present in the plant are known to have potent pharmacological properties, which could be responsible for its inhibitory activity. The IC_50_ value of the plant was slightly lower than the positive control suggesting that *A. tuberculata* could be a potential alternative to existing therapeutic agents. This inhibition of DPP‐4 activity can lead to increased concentrations of GLP‐1 and GIP in the body, which can improve glucose metabolism and help control blood sugar levels. In addition to the direct inhibition of DPP‐IV, some plant metabolites also possess other actions relevant to diabetes, such as stimulation of glucose‐stimulated insulin secretion, inhibition of alpha‐amylase, activation of adenosine monophosphate‐activated protein kinase (AMPK), and modulation of the expression of genes involved in glucose metabolism (Shehadeh et al. 2021). Furthermore, DPP‐4 inhibitors have anti‐inflammatory and antioxidant effects as well, which may be beneficial for patients with diabetes (Shastri and Gadhave 2022). In summary, plant metabolites have been shown to act as DPP‐4 inhibitors and can help to improve glucose metabolism and reduce the risk of complications associated with diabetes which are further validated through molecular docking.

### 3.6 Molecular Docking and Protein‐Ligand Interaction Analysis

**FIGURE 5:**
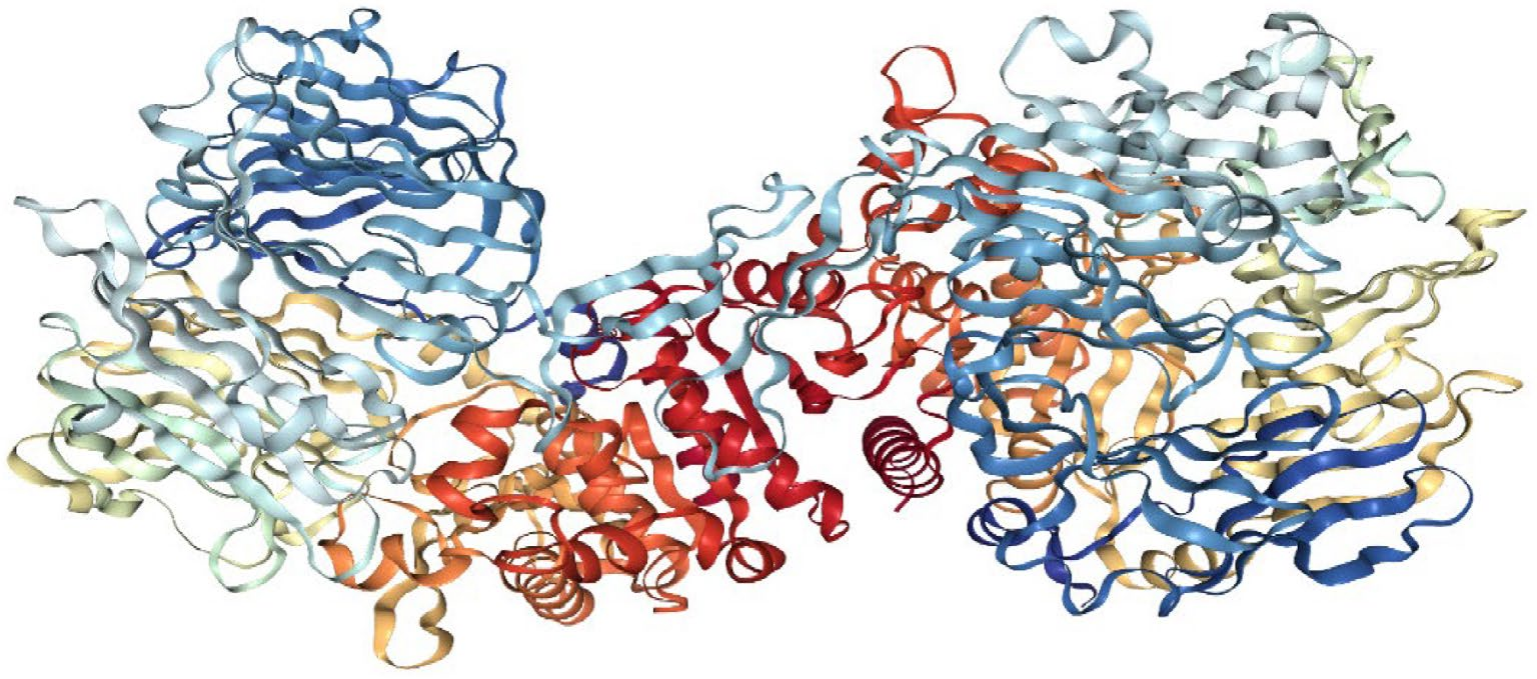
Three‐dimensional structure of dipeptidyl peptidase‐4 (DPP‐IV) protein [PDB accession ID: 5I7U].

Target protein and chemical compounds are the requirements to start the molecular docking procedure. Experimentally identified 24 compounds from *A. tuberculata* were used as a dataset for molecular docking studies. The two‐dimensional structure of the selected 24 compounds was drawn very carefully by ChemDraw Ultra 12.0 software (Table S3) (Cousins 2011). The three‐dimensional structure of the selected targeted protein, DPP‐4 protein, was retrieved from the protein data bank (accession ID 5I7U) with resolution 1.95 Å (Figure 5) (Wu et al. 2016). These 24 extracted natural compounds from *A. tuberculata* were expected to have anti‐DM potential so it was validated by the application of computer‐aided drug design procedures, such as the calculation of protein‐ligand binding interaction behavior with the DPP‐4 protein target, which is one of the novel therapeutic agents to design potential drugs for DM. Several FDA‐approved DPP‐4 inhibitors are available in the market and used for the treatment of DM.

Selected dataset of 24 chemical compounds extracted from *A. tuberculata* used for molecular docking analysis has several biological activities, while in this study we focused on the activities of chemical compounds as DPP‐4 inhibitors which could be significant anti‐DM agents. Isorhamnetin could improve glycemic levels in DM patients and its related complications such as diabetic cataracts, hypertension, obesity, lipid peroxidation, and hyperglycemia (Li et al. 2016). Bilobalide is significant for the treatment of heart disease and DM; it is also a therapeutic agent for inflammation and insulin resistance to treat the complications of DM (Lim and Park 2013). While extensive research is required to confirm the Ginkgolide J activity as an anti‐DM agent (Wang et al. 2006). Kaempferol‐7‐*O*‐glucoside can improve insulin resistance and downregulation of glucose in blood and cholesterol profile in Type 2 DM (Nishina et al. 2019). Quercetin‐3‐*O*‐glucoside can mediate the various metabolic activities, ameliorate the DM conditions, stimulate the insulin signaling pathway, and improve the lipid peroxidation in tissues (Panda and Kar 2007). The extract of *A. tuberculata* is used for DM treatment; thus, it is required to check the properties of Kaempferol‐7‐O‐rutinoside chemical compound as an anti‐DM drug (Abdel‐Sattar et al. 2007). Quercetin‐3‐*O*‐rutinoside is an important phenolic glycoside with various medicinal properties; it also includes anti‐DM (Nazir et al. 2017).

Further research is required to confirm its mechanism of action for DM. Isorhamnetin‐3‐*O*‐rutinoside is also a beneficial phenolic glycoside and useful dietary supplement and anti‐DM agent (Jia et al. 2019). Extensive research is still required to understand its mechanism of action for DM. Russelioside C could significantly improve the fasting serum glucose and glycated hemoglobin and insulin sensitivity, and serum insulin and cholesterol profile in DM patients. Previous studies provide evidence of its use as an anti‐diabetic and anti‐hyperglycemic agent. Pregnane glycoside is useful to manage hyperglycemia in DM patients (Abdel‐Sattar et al. 2016). Russelioside G is a pregnane glycoside, its diabetic properties are required to be investigated. Digoxin is usually prescribed to DM patients with heart‐related complications but its mechanism of action concerning DM complications is necessary to be researched. Ginkgolide C is an active extract, which is used for various medicinal aspects, but its anti‐DM activities are required to be investigated (Wang et al. 2006). Pregnane glycosides are useful for diabetic treatment. Bouceroside—ADC, Russelioside F, and Russelioside E are pregnane glycosides, its DM properties are required to be investigated.

DPP‐4 protein shares several names such as adenosine deaminase complex protein 2 and CD26 protein also, encoded by DPP‐4 gene, related to various biological activities such regulation of insulin signaling pathway, immune regulatory pathway, apoptosis, tumor suppression, and progression (Tanwar et al. 2014). In the case of diabetes mellitus, DPP‐4 inhibition is beneficial to control hyperglycemia and improve insulin secretion and an additional benefit is there is no weight gain with control of lipid profile. Several approaches have been used to investigate the activities of the selected compounds previously, while we have developed the computer‐aided drug design approach to analyze the binding behavior of DDP‐IV protein with the selected compounds to validate its activity as anti‐DM agents and also predicted their pharmacological behavior which could be helpful to understand the chemical nature of extracted 24 compounds when put forward to the drug development phase. A summary of the docking results of the selected 24 compounds is shown in Table S5, each compound is analyzed in detail concerning its binding energy, and its hydrophobic and hydrogen bonding interactions.

**FIGURE 6:**
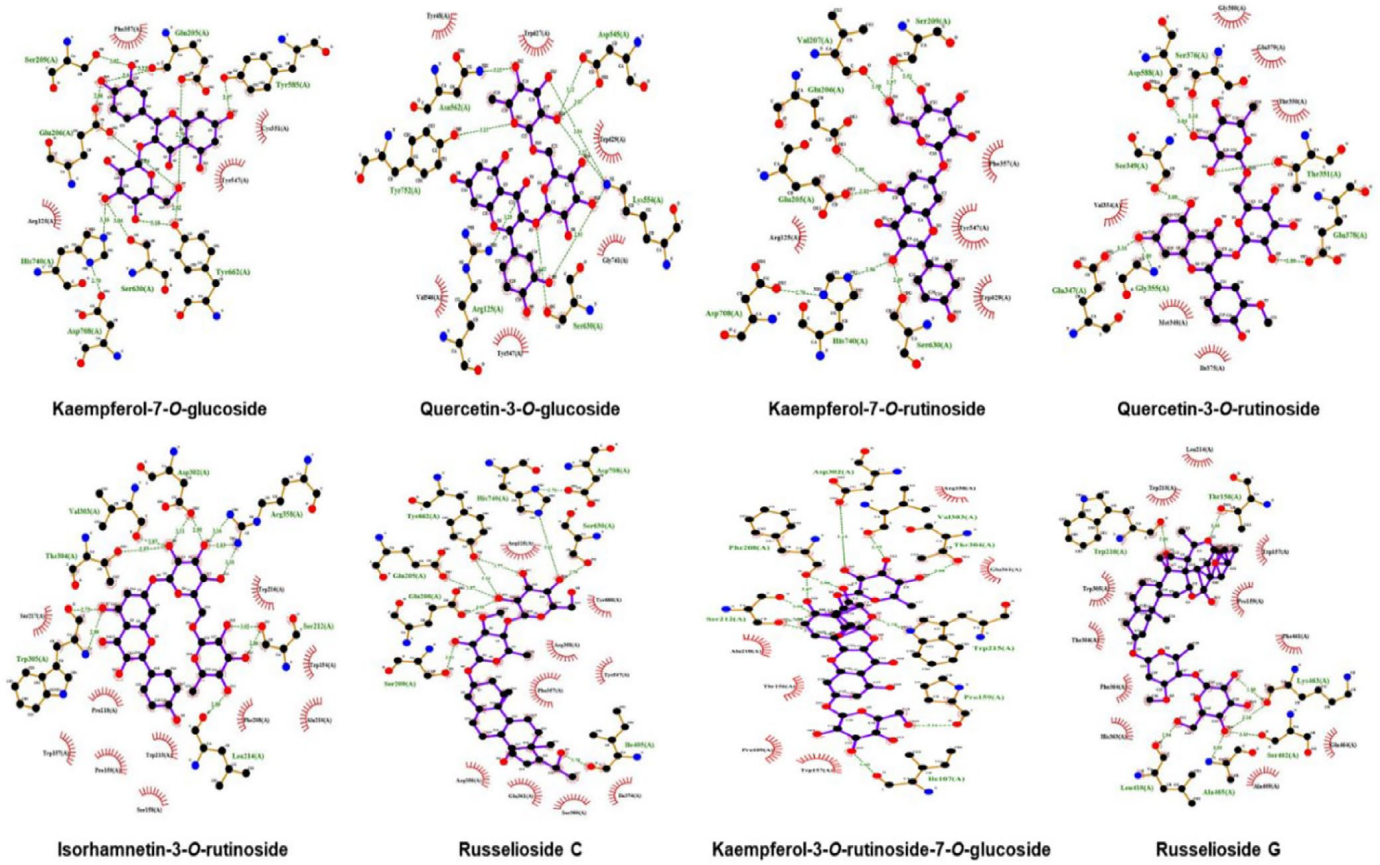
It shows molecular structures. Two‐dimensional representation of the best‐docked complex of the selected dataset of chemical compounds within the binding pocket of the dipeptidyl peptidase‐4 protein. Hydrophobic residues are highlighted in red while green indicates residues forming hydrogen bonds within 4A of the active binding pocket of DPP‐IV.

**FIGURE 7:**
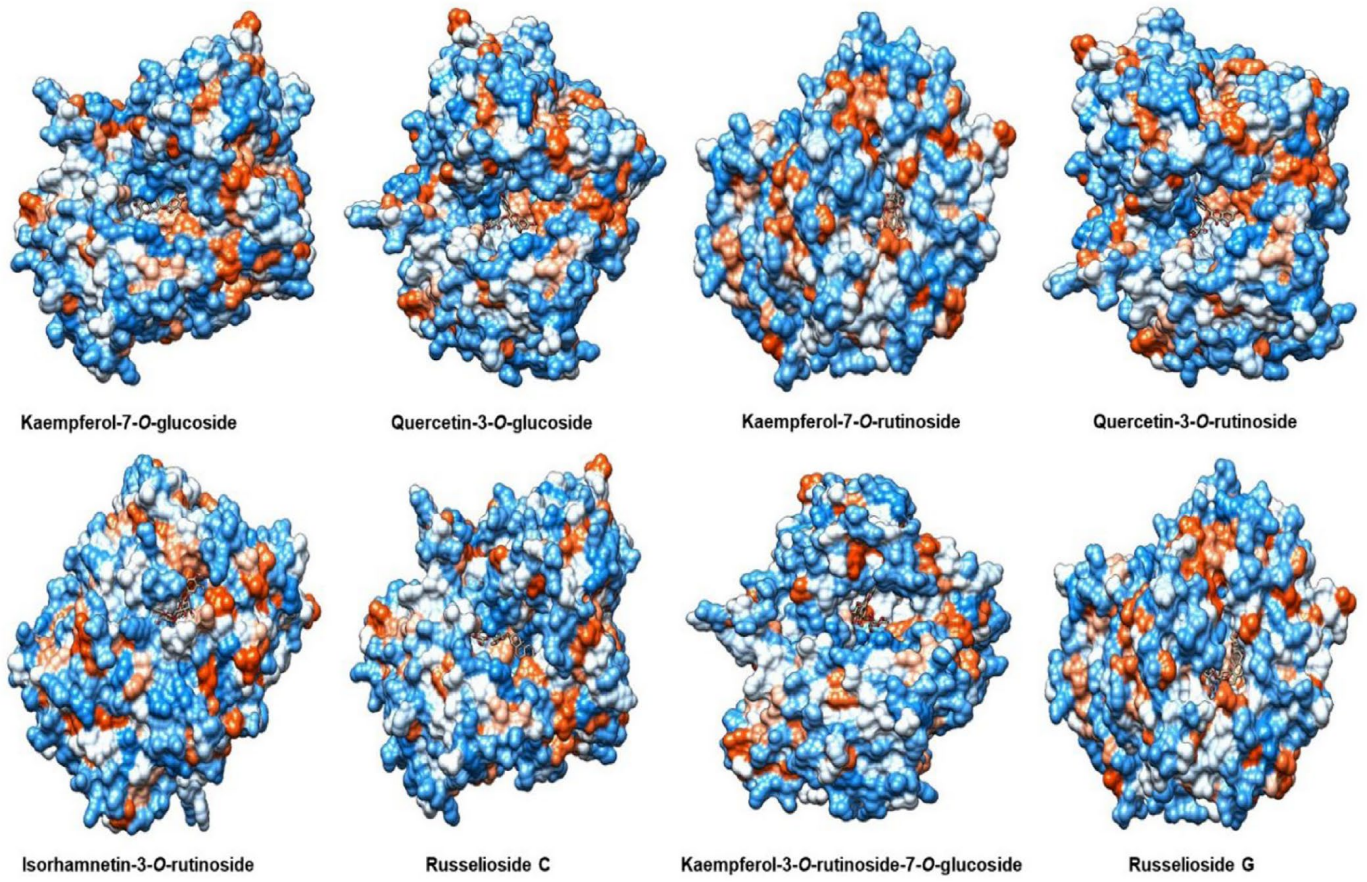
It shows a three‐dimensional representation of the best‐docked complex of the selected dataset of chemical compounds within the binding pocket of the dipeptidyl peptidase‐4 protein.

Protein‐ligand binding interactions within the best pose with low energy models and more number of interactions were considered to be the best‐docked complex visualized by Ligplot software (Wallace et al. 1995), hydrophobic and hydrogen bonding residues are shown in the two‐dimensional plots, red spikes showing how the hydrophobic residues and green colored residues are making hydrogen bonds with the chemical compound within the vicinity of 4A in the active binding pocket of the target protein while the three‐dimensional representation of ligand poses within the hydrophobic pocket is generated by Chimera software (Figures 6 and 7) (Pettersen et al. 2004). DPP‐4 protein is hydrophobic, so most of the active pockets make hydrophobic interactions with the chemical compounds bonded to it.

After the application of molecular docking, it clearly understood the DPP‐4 inhibitory nature of each selected compound, and nine compounds demonstrated very significant modes of binding interactions within the binding pocket of the target protein. Kaempferol‐7‐O‐rutinoside and Kaempferol‐3‐O‐rutinoside‐7‐O‐glucoside show the best binding mode of interaction with DPP4 target protein presenting 12 hydrogen bonds, 38 hydrophobic bonds with −9.4 binding energy score while Quercetin‐3‐O‐glucoside also presented significant docking results with 11 hydrogen bonds, 27 hydrophobic bonds with −7.8 binding energy score, Quercetin‐3‐O‐rutinoside presented 9 hydrogen bonds and 37 hydrophobic bonds with −8.8 binding energy score, Russelioside C 8 hydrogen bonds and 41 hydrophobic bonds with −8.9 binding energy score, Russelioside G 7 hydrogen bonds and 36 hydrophobic bonds with −10.3 binding energy score, Bouceroside–ADC 7 hydrogen bonds and 37 hydrophobic bonds with −9.8 binding energy score, Isorhamnetin‐3‐O‐rutinoside 7 hydrogen bonds and 53 hydrophobic bonds with −8.9 binding energy score, and Kaempferol‐7‐O‐glucoside 7 hydrogen bonds and 27 hydrophobic bonds with −8.2 binding energy score, two dimensional and three dimensional.

### 3.7 Pharmacological Prediction

Pharmacological estimations account for the ADMET properties of drugs in the initial phase of development (Ntie‐Kang et al. 2013), but in the case of natural products mostly some properties are not fulfilled, as for the significant information helpful for the future synthesis and drug development. It is preferred to calculate the pharmacological properties in this study. Table 2 presents the pharmacological predictions in terms of drug‐likeness, and toxicity estimations concerning mutagenic, tumorigenic, reproductive, and irritant effects, pharmacokinetics, water solubility, and indications beneficial for medicinal chemistry synthesis of chemical compounds. As from the selected 24 compounds extracted from *A. tuberculata*, usually, everyone does not fulfill the drug‐like properties, such as Lipinski rule (Lipinski et al. 2012) and Veber rule (Veber et al. 2002) because natural compounds consist of different heavy atoms and functional groups, so its weight is generally more than 500 Da, but natural compounds are highly active and previously proved their medicinal status in terms of important biological activities (Newman and Cragg 2020). Bilobalide, Ginkgolide J, Quercetin‐3‐O‐glucoside, Caratuberside E, Quercetin‐3‐*O*‐rutinoside, Isorhamnetin‐3‐*O*‐rutinoside, Digoxin, Ginkgolide C, Caratuberside E, Isopropenyl Glycopyranosides, Tragopogonsaponin M has presented no toxic effects in terms of mutagenicity, tumorigenicity, reproductive and irritant effects. Pharmacokinetics profile of each compound is evaluated to confirm the bioavailability of chemical compounds such as GI absorption, BBB permeability, P‐gp substrate, CYP1A2 inhibitor, CYP2C19 inhibitor, CYP2C9 inhibitor, CYP2D6 inhibitor, CYP3A4 inhibitor, Log Kp value for skin permeability, and water solubility estimation.

**TABLE 2:** Pharmacological prediction of selected dataset of chemical compounds.

Chemical compounds	Drug‐likeness	Toxicity estimation	Pharmacokinetics	Water solubility	Medicinal chemistry
Deoxyguanosine	Lipinski = No violation Veber = No violation Bioavailability =0.55	Mutagenic effects = high Tumorigenic effects = high Reproductive effects = high Irritant effects = high	GI absorption = high BBB permeant = No P‐gp substrate = No CYP1A2inhibitor = No CYP2C19 inhibitor = No CYP2C9 inhibitor = No CYP2D6 inhibitor = No CYP3A4 inhibitor = No Log Kp (skin permeation) = −7.16 cm/s	Highly soluble	PAINS = No alert Break = One alert Synthetic accessibility = 2.08
Isorhamnetin	Lipinski = No violation Veber = No violation Bioavailability =0.55	Mutagenic effects = high Tumorigenic effects = none Reproductive effects = none Irritant effects = none	GI absorption = high BBB permeant = No P‐gp substrate = No CYP1A2inhibitor = Yes CYP2C9 inhibitor = No CYP2C9 inhibitor = No CYP2D6 inhibitor = Yes CYP3A4 inhibitor = Yes Log Kp (skin permeation) = −6.90 cm/s	Soluble	PAINS = No alert Break = No alert Synthetic accessibility = 3.26
Bilobalide	Lipinski = No violation Veber = No violation Bioavailability =0.55	Mutagenic effects = none Tumorigenic effects = none Reproductive effects = none Irritant effects = none	GI absorption = high BBB permeant = No P‐gp substrate = Yes CYP1A2inhibitor = No CYP2C9 inhibitor = No CYP2C9 inhibitor = No CYP2D6 inhibitor = No CYP3A4 inhibitor = No Log Kp (skin permeation) = −8.48 cm/s	Highly soluble	PAINS = No alert Break = One alert Synthetic accessibility = 5.41
Ginkgolide J	Lipinski = No violation Veber = One violation Bioavailability =0.55	Mutagenic effects = none Tumorigenic effects = none Reproductive effects = none Irritant effects = none	GI absorption = Low BBB permeant = No P‐gp substrate = Yes CYP1A2inhibitor = No CYP2C9 inhibitor = No CYP2C9 inhibitor = No CYP2D6 inhibitor = No CYP3A4 inhibitor = No Log Kp (skin permeation) = −9.16 cm/s	Soluble	PAINS = No alert Break = One alert Synthetic accessibility = 6.39
Kaempferol‐7‐*O*‐glucoside	Lipinski = Two violations Veber = One violation Bioavailability =0.17	Mutagenic effects = high Tumorigenic effects = none Reproductive effects = none Irritant effects = none	GI absorption = Low BBB permeant = No P‐gp substrate = No CYP1A2inhibitor = No CYP2C9 inhibitor = No CYP2C9 inhibitor = No CYP2D6 inhibitor = No CYP3A4 inhibitor = No Log Kp (skin permeation) = −8.52 cm/s	Soluble	PAINS = No alert Break = No alert Synthetic accessibility = 5.24
Quercetin‐3‐O‐glucoside	Lipinski = Two violations Veber = One violation Bioavailability =0.17	Mutagenic effects = none Tumorigenic effects = none Reproductive effects = none Irritant effects = none	GI absorption = Low BBB permeant = No P‐gp substrate = No CYP1A2inhibitor = No CYP2C9 inhibitor = No CYP2C9 inhibitor = No CYP2D6 inhibitor = No CYP3A4 inhibitor = No Log Kp (skin permeation) = −8.88 cm/s	Soluble	PAINS = One alert Break = One alert Synthetic accessibility = 5.32
Caratuberside E	Lipinski = Two violations Veber = Two violations Bioavailability =0.17	Mutagenic effects = none Tumorigenic effects = none Reproductive effects = none Irritant effects = none	GI absorption = Low BBB permeant = No P‐gp substrate = Yes CYP1A2inhibitor = No CYP2C9 inhibitor = No CYP2C9 inhibitor = No CYP2D6 inhibitor = No CYP3A4 inhibitor = No Log Kp (skin permeation) = −9.18 cm/s	Moderately soluble	PAINS = No alert Break = Two alerts Synthetic accessibility = 9.46
Kaempferol‐7‐*O*‐rutinoside	Lipinski = Three violations Veber = One violation Bioavailability =0.17	Mutagenic effects = high Tumorigenic effects = none Reproductive effects = none Irritant effects = none	GI absorption = Low BBB permeant = No P‐gp substrate = Yes CYP1A2inhibitor = No CYP2C9 inhibitor = No CYP2C9 inhibitor = No CYP2D6 inhibitor = No CYP3A4 inhibitor = No Log Kp (skin permeation) = −9.91 cm/s	Soluble	PAINS = No alert Break = No alert Synthetic accessibility = 6.43
Quercetin‐3‐*O*‐rutinoside	Lipinski = Three violations Veber = One violation Bioavailability =0.17	Mutagenic effects = none Tumorigenic effects = none Reproductive effects = none Irritant effects = none	GI absorption = Low BBB permeant = No P‐gp substrate = Yes CYP1A2inhibitor = No CYP2C9 inhibitor = No CYP2C9 inhibitor = No CYP2D6 inhibitor = No CYP3A4 inhibitor = No Log Kp (skin permeation) = −10.26 cm/s	Soluble	PAINS = One alert Break = One alert Synthetic accessibility = 6.52
Isorhamnetin‐3‐*O*‐rutinoside	Lipinski = Three violations Veber = One violation Bioavailability =0.17	Mutagenic effects = none Tumorigenic effects = none Reproductive effects = none Irritant effects = none	GI absorption = Low BBB permeant = No P‐gp substrate = Yes CYP1A2inhibitor = No CYP2C9 inhibitor = No CYP2C9 inhibitor = No CYP2D6 inhibitor = No CYP3A4 inhibitor = No Log Kp (skin permeation) = −10.12 cm/s	Soluble	PAINS = No alert Break = No alert Synthetic accessibility = 6.64
Russelioside C	Lipinski = Three violations Veber = One violation Bioavailability =0.17	Mutagenic effects = none Tumorigenic effects = none Reproductive effects = low Irritant effects = none	GI absorption = Low BBB permeant = No P‐gp substrate = Yes CYP1A2inhibitor = No CYP2C9 inhibitor = No CYP2C9 inhibitor = No CYP2D6 inhibitor = No CYP3A4 inhibitor = No Log Kp (skin permeation) = −9.75 cm/s	Soluble	PAINS = No alert Break = One alert Synthetic accessibility = 8.21
Kaempferol‐3‐*O*‐rutinoside‐7‐*O*‐glucoside	Lipinski = Three violations Veber = four violation Bioavailability =0.17	Mutagenic effects = high Tumorigenic effects = none Reproductive effects = none Irritant effects = none	GI absorption = Low BBB permeant = No P‐gp substrate = Yes CYP1A2inhibitor = No CYP2C9 inhibitor = No CYP2C9 inhibitor = No CYP2D6 inhibitor = No CYP3A4 inhibitor = No Log Kp (skin permeation) = −12.18 cm/s	Soluble	PAINS = No alert Break = No alert Synthetic accessibility = 7.65
Russelioside G	Lipinski = Two violations Veber = two violation Bioavailability =0.17	Mutagenic effects = none Tumorigenic effects = none Reproductive effects = low Irritant effects = none	GI absorption = Low BBB permeant = No P‐gp substrate = Yes CYP1A2inhibitor = No CYP2C9 inhibitor = No CYP2C9 inhibitor = No CYP2D6 inhibitor = No CYP3A4 inhibitor = No Log Kp (skin permeation) = −8.78 cm/s	Soluble	PAINS = No alert Break = Two alert Synthetic accessibility = 8.44
Digoxin	Lipinski = Three violations Veber = One violation Bioavailability =0.17	Mutagenic effects = none Tumorigenic effects = none Reproductive effects = none Irritant effects = none	GI absorption = Low BBB permeant = No P‐gp substrate = Yes CYP1A2inhibitor = No CYP2C9 inhibitor = No CYP2C9 inhibitor = No CYP2D6 inhibitor = No CYP3A4 inhibitor = No Log Kp (skin permeation) = −10.78 cm/s	Moderately soluble	PAINS = No alert Break = One alert Synthetic accessibility = 8.81
Russelioside B	Lipinski = Three violations Veber = Four violation Bioavailability =0.17	Mutagenic effects = none Tumorigenic effects = none Reproductive effects = low Irritant effects = none	GI absorption = Low BBB permeant = No P‐gp substrate = Yes CYP1A2inhibitor = No CYP2C9 inhibitor = No CYP2C9 inhibitor = No CYP2D6 inhibitor = No CYP3A4 inhibitor = No Log Kp (skin permeation) = −11.87 cm/s	Soluble	PAINS = No alert Break = One alert Synthetic accessibility = 9.27
Ginkgolide C	Lipinski = One violations Veber = One violation Bioavailability =0.55	Mutagenic effects = none Tumorigenic effects = none Reproductive effects = none Irritant effects = none	GI absorption = Low BBB permeant = No P‐gp substrate = Yes CYP1A2inhibitor = No CYP2C9 inhibitor = No CYP2C9 inhibitor = No CYP2D6 inhibitor = No CYP3A4 inhibitor = No Log Kp (skin permeation) = −9.95 cm/s	Soluble	PAINS = No alert Break = One alert Synthetic accessibility = 6.48
Pregnane glycoside	Lipinski = two violations Veber = Three violation Bioavailability =0.17	Mutagenic effects = none Tumorigenic effects = none Reproductive effects = low Irritant effects = high	GI absorption = Low BBB permeant = No P‐gp substrate = Yes CYP1A2inhibitor = No CYP2C9 inhibitor = No CYP2C9 inhibitor = No CYP2D6 inhibitor = No CYP3A4 inhibitor = No Log Kp (skin permeation) = −8.18 cm/s	Soluble	PAINS = No alert Break = Three alert Synthetic accessibility = 8.80
Bouceroside – ADC	Lipinski = Two violations Veber = Two violation Bioavailability =0.17	Mutagenic effects = none Tumorigenic effects = none Reproductive effects = low Irritant effects = none	GI absorption = Low BBB permeant = No P‐gp substrate = No CYP1A2inhibitor = No CYP2C9 inhibitor = No CYP2C9 inhibitor = No CYP2D6 inhibitor = No CYP3A4 inhibitor = No Log Kp (skin permeation) = −8.77 cm/s	Moderately soluble	PAINS = No alert Break = One alert Synthetic accessibility = 9.41
Russelioside F	Lipinski = Two violations Veber = Two violation Bioavailability =0.17	Mutagenic effects = none Tumorigenic effects = none Reproductive effects = low Irritant effects = none	GI absorption = Low BBB permeant = No P‐gp substrate = Yes CYP1A2inhibitor = No CYP2C9 inhibitor = No CYP2C9 inhibitor = No CYP2D6 inhibitor = No CYP3A4 inhibitor = No Log Kp (skin permeation) = −9.37 cm/s	Soluble	PAINS = No alert Break = Two alert Synthetic accessibility = 9.55
Caratuberside E	Lipinski = Two violations Veber = Two violation Bioavailability =0.17	Mutagenic effects = none Tumorigenic effects = none Reproductive effects = none Irritant effects = none	GI absorption = Low BBB permeant = No P‐gp substrate = Yes CYP1A2inhibitor = No CYP2C9 inhibitor = No CYP2C9 inhibitor = No CYP2D6 inhibitor = No CYP3A4 inhibitor = No Log Kp (skin permeation) = −9.35 cm/s	Moderately soluble	PAINS = No alert Break = Two alert Synthetic accessibility = 9.63
Raratuberside C	Lipinski = Two violations Veber = Two violation Bioavailability =0.17	Mutagenic effects = none Tumorigenic effects = none Reproductive effects = low Irritant effects = high	GI absorption = Low BBB permeant = No P‐gp substrate = Yes CYP1A2inhibitor = No CYP2C9 inhibitor = No CYP2C9 inhibitor = No CYP2D6 inhibitor = No CYP3A4 inhibitor = No Log Kp (skin permeation) = −9.53 cm/s	Moderately soluble	PAINS = No alert Break = Two alert Synthetic accessibility = 9.79
Isopropenyl Glycopyranosides	Lipinski = Two violations Veber = Two violation Bioavailability =0.17	Mutagenic effects = none Tumorigenic effects = none Reproductive effects = none Irritant effects = none	GI absorption = Low BBB permeant = No P‐gp substrate = Yes CYP1A2inhibitor = No CYP2C9 inhibitor = No CYP2C9 inhibitor = No CYP2D6 inhibitor = No CYP3A4 inhibitor = Yes Log Kp (skin permeation) = −5.61 cm/s	Insoluble	PAINS = No alert Break = One alert Synthetic accessibility = 8.89
Tragopogonsaponin M	Lipinski = Three violations Veber = Three violation Bioavailability =0.17	Mutagenic effects = none Tumorigenic effects = none Reproductive effects = none Irritant effects = none	GI absorption = Low BBB permeant = No P‐gp substrate = Yes CYP1A2inhibitor = No CYP2C9 inhibitor = No CYP2C9 inhibitor = No CYP2D6 inhibitor = No CYP3A4 inhibitor = No Log Kp (skin permeation) = −8,07 cm/s	Poorly soluble	PAINS = No alert Break = two alert Synthetic accessibility = 9.33
12,20‐di‐*O*‐benzoyl boucerin	Lipinski = Two violations Veber = One violation Bioavailability =0.17	Mutagenic effects = none Tumorigenic effects = low Reproductive effects = low Irritant effects = none	GI absorption = Low BBB permeant = No P‐gp substrate = No CYP1A2inhibitor = No CYP2C9 inhibitor = No CYP2C9 inhibitor = No CYP2D6 inhibitor = No CYP3A4 inhibitor = No Log Kp (skin permeation) = −8.36 cm/s	Poorly soluble	PAINS = No alert Break = One alert Synthetic accessibility = 9.61

Log Kp values are acceptable for the selected 24 compounds; most of them are soluble in nature, and medicinal chemistry properties are calculated to highlight problematic fragments of chemical compounds, such as PAINS and break alerts, which indicate the highly reactive functional groups that could be prerequisites for the synthesis of these chemical compounds in the laboratory so each compound could be optimized to manage these alerts and make the drug orally bioavailable without any harmful effects, and the synthetic accessibility score depends on each structural fragment of the chemical compound and is helpful for the easy synthesis of chemicals in the laboratory. Out of 24 selected chemical compounds, Isorhamnetin, Kaempferol‐7‐O‐glucoside, Kaempferol‐7‐O‐rutinoside, Isorhamnetin‐3‐O‐rutinoside, and Kaempferol‐3‐O‐rutinoside‐7‐O‐glucoside have demonstrated no PAINS and break alerts, while other chemical compounds have some indications for the medicinal chemist to research and optimize these chemical compounds in the laboratory to deliver a safe drug to market.

## 4 Conclusion

In conclusion, this study has demonstrated the potential antidiabetic activity of *A. tuberculata* and identified new phytochemicals with a high antidiabetic potential. The phytochemical analysis revealed the presence of various classes of glycosides, flavonoid glycosides, flavonols, cardiac glycosides, and triterpenoids which are responsible for the antioxidant and antidiabetic activity of the plant. The results of in vitro DPP‐4 assay, molecular docking, and pharmacological prediction studies agreed with the findings that kaempferol‐7‐O‐rutinoside and kaempferol‐3‐O‐rutinoside‐7‐O‐glucoside have the potential to be used as antidiabetic agents. Out of 24 selected chemical compounds, Isorhamnetin, Kaempferol‐7‐O‐glucoside, Kaempferol‐7‐O‐rutinoside, Isorhamnetin‐3‐O‐rutinoside, Kaempferol‐3‐O‐rutinoside‐7‐O‐glucoside, has demonstrated no PAINS, and Break alerts, indicating the safe use of these compounds for medical purpose. The untargeted metabolomics analysis revealed that *A. tuberculata* has a wide range of bioactive secondary metabolites, which can be further studied to develop new antidiabetic drugs. Additionally, the antioxidant activity of *A. tuberculata* could be beneficial in the prevention of obesity and diabetes‐related complications. Further, in vivo studies are needed to establish the safety, efficacy, and pharmacokinetic properties of the compounds for potential therapeutic use.

## Author Contributions

**Ilham Khan:** conceptualization (equal), methodology (equal), writing – original draft (equal). **Shabana Bibi:** data curation (equal), formal analysis (equal), investigation (equal), writing – original draft (equal). **Junaid Shehzad:** investigation (equal), writing – original draft (equal). **Zarqa Riaz:** software (equal), validation (equal), writing – original draft (equal). **Muhammad Saad Khan:** formal analysis (equal), software (equal), writing – original draft (equal). **Mansour Ghorbanpour:** writing – original draft (equal). **Murtaza Hasan:** resources (equal). **Ghazala Mustafa:** conceptualization (equal), resources (equal), supervision (equal).

## Ethics Statement

The authors have nothing to report.

## Consent

The authors agreed to publish this work. All the authors agreed to participate in this work.

## Conflicts of Interest

The authors declare no conflicts of interest.

## Supporting information


Data S1.

## Data Availability

The data and materials will be available on request.

## References

[fsn370494-bib-0002] Abdel‐Sattar, E. A. , H. M. Abdallah , A. Khedr , A. B. Abdel‐Naim , and I. A. Shehata . 2013. “Antihyperglycemic Activity of Caralluma Tuberculata in Streptozotocin‐Induced Diabetic Rats.” Food and Chemical Toxicology 59: 111–117. 10.1016/j.fct.2013.05.060. 23770343

[fsn370494-bib-0001] Abdel‐Sattar, E. , A. A. Ahmed , M. E. Hegazy , M. A. Farag , and M. A. Al‐Yahya . 2007. “Acylated Pregnane Glycosides From Caralluma Russeliana.” Phytochemistry 68, no. 10: 1459–1463. 10.1016/j.phytochem.2007.03.009. 17449076

[fsn370494-bib-0005] Abdel‐Sattar, E. , F. M. Harraz , S. A. Ghareib , A. A. Elberry , S. Gabr , and M. I. Suliaman . 2011. “Antihyperglycaemic and Hypolipidaemic Effects of the Methanolic Extract of Caralluma Tuberculata in Streptozotocin‐Induced Diabetic Rats.” Natural Product Research 25, no. 12: 1171–1179. 10.1080/14786419.2010.490782. 21740282

[fsn370494-bib-0004] Abdel‐Sattar, E. , F. M. Harraz , S. M. A. Al‐ansari , et al. 2008. “Acylated Pregnane Glycosides From Caralluma Tuberculata and Their Antiparasitic Activity.” Phytochemistry 69, no. 11: 2180–2186. 10.1016/j.phytochem.2008.05.017. 18614190

[fsn370494-bib-0003] Abdel‐Sattar, E. , S. A. El‐Maraghy , R. S. El‐Dine , and S. M. Rizk . 2016. “Russelioside B, a Pregnane Glycoside Ameliorates Hyperglycemia in Streptozotocin Induced Diabetic Rats by Regulating Key Enzymes of Glucose Metabolism.” Chemico‐Biological Interactions 252: 47–53. 10.1016/j.cbi.2016.03.033. 27038876

[fsn370494-bib-0006] Ablajan, K. , and A. Tuoheti . 2013. “Fragmentation Characteristics and Isomeric Differentiation of Flavonol O‐Rhamnosides Using Negative Ion Electrospray Ionization Tandem Mass Spectrometry.” Rapid Communications in Mass Spectrometry 27, no. 3: 451–460. 10.1002/rcm.6476. 23280977

[fsn370494-bib-0007] Al‐Fatimi, M. 2019. “Ethnobotanical Survey of Medicinal Plants in Central Abyan Governorate, Yemen.” Journal of Ethnopharmacology 241: 111973. 10.1016/j.jep.2019.111973. 31146001

[fsn370494-bib-0008] Antar, S. A. , N. A. Ashour , M. Sharaky , et al. 2023. “Diabetes Mellitus: Classification, Mediators, and Complications; A Gate to Identify Potential Targets for the Development of New Effective Treatments.” Biomedicine and Pharmacotherapy 168: 115734. 37857245 10.1016/j.biopha.2023.115734

[fsn370494-bib-0009] Arshad, H. , and G. Mustafa . 2023. “Hormonal Response in Plants Influenced by Reactive Oxygen Species.” In Reactive Oxygen Species: Prospects in Plant Metabolism. Springer Nature.

[fsn370494-bib-0011] Centers for Disease Control and Prevention . 2014. National Diabetes Statistics Report: Estimates of Diabetes and Its Burden in the United States. US Department of Health and Human Services.

[fsn370494-bib-0012] Chandran, R. , T. Sajeesh , and T. Parimelazhagan . 2014. “Total Phenolic Content, Anti‐Radical Property and HPLC Profiles of Caralluma Diffusa (Wight) N.E. Br.” Journal of Biologically Active Products From Nature 4, no. 3: 188–195. 10.1080/22311866.2014.933082.

[fsn370494-bib-0013] Cousins, K. R. 2011. “Computer Review of ChemDraw Ultra 12.0.” Journal of the American Chemical Society 133, no. 21: 8388. 21561109 10.1021/ja204075s

[fsn370494-bib-0014] Daina, A. , O. Michielin , and V. Zoete . 2017. “SwissADME: A Free Web Tool to Evaluate Pharmacokinetics, Drug‐Likeness and Medicinal Chemistry Friendliness of Small Molecules.” Scientific Reports 7: 42717. 10.1038/srep42717. 28256516 PMC5335600

[fsn370494-bib-0015] De Vos, R. C. H. , S. Moco , A. Lommen , J. J. B. Keurentjes , R. J. Bino , and R. D. Hall . 2007. “Untargeted Large‐Scale Plant Metabolomics Using Liquid Chromatography Coupled to Mass Spectrometry.” Nature Protocols 2, no. 4: 778–791. 10.1038/nprot.2007.95. 17446877

[fsn370494-bib-0016] Elshafie, H. S. , I. Camele , and A. A. Mohamed . 2023. “A Comprehensive Review on the Biological, Agricultural and Pharmaceutical Properties of Secondary Metabolites Based‐Plant Origin.” International Journal of Molecular Sciences 24, no. 4: 3266. 36834673 10.3390/ijms24043266 PMC9959544

[fsn370494-bib-0017] Farag, R. S. , M. S. Abdel‐Latif , H. H. Abd , E. Baky , and L. S. Tawfeek . 2020. “Phytochemical Screening and Antioxidant Activity of Some Medicinal Plants' Crude Juices.” Biotechnology Reports 28: e00536. 10.1016/j.btre.2020.e00536. 33088732 PMC7559852

[fsn370494-bib-0018] Formisano, C. , F. Senatore , G. Della Porta , et al. 2009. “Headspace Volatile Composition of the Flowers of Caralluma Europaea n.e.Br. (Apocynaceae).” Molecules 14, no. 11: 4597–4613. 10.3390/molecules14114597. 19924088 PMC6254730

[fsn370494-bib-0020] Hasan, M. , H. Gulzar , A. Zafar , et al. 2021. “Multiplexing Surface Anchored Functionalized Iron Carbide Nanoparticle: A Low Molecular Weight Proteome Responsive Nano‐Tracer.” Colloids and Surfaces B: Biointerfaces 203: 111746. 10.1016/j.colsurfb.2021.111746. 33839473

[fsn370494-bib-0021] Hasan, M. , K. Mehmood , G. Mustafa , et al. 2021. “Phytotoxic Evaluation of Phytosynthesized Silver Nanoparticles on Lettuce.” Coatings 11: 225. 10.3390/coatings11020225.

[fsn370494-bib-0019] Hasan, M. , R. Dai , B. Asghari , et al. 2014. “Enhancement of Rice Growth and Production of Growth‐Promoting Phytohormones by Inoculation With Rhizobium and Other Rhizobacteria.” World Applied Sciences Journal 31, no. 10: 1734–1743.

[fsn370494-bib-0022] Hasan, M. , S. Rafique , A. Zafar , et al. 2020. “Physiological and Anti‐Oxidative Response of Biologically and Chemically Synthesized Iron Oxide: *Zea Mays* A Case Study.” Heliyon 6: e04595. 10.1016/j.heliyon.2020.e04595. 32923707 PMC7475124

[fsn370494-bib-0023] Hasan, M. , T. Tariq , G. Mustafa , E. A. A. Ismail , F. A. Awwad , and M. Hatami . 2024. “Biogenic Zinc Oxide Nanoregulator Determines the Quantitative Analysis of Morpho‐Anatomical and Antioxidant Capacity in *Lactuca Sativa* L.” Food Science & Nutrition 12: 7954–7967. 10.1002/fsn3.4261. 39479692 PMC11521655

[fsn370494-bib-0024] He, J. H. , L. X. Chen , and H. Li . 2019. “Progress in the Discovery of Naturally Occurring Anti‐Diabetic Drugs and in the Identification of Their Molecular Targets.” Fitoterapia 134: 270–289. 30840917 10.1016/j.fitote.2019.02.033

[fsn370494-bib-0026] Iqbal, J. , W. Li , K. Ullah , et al. 2013. “Study of Rat Hypothalamic Proteome by HPLC/ESI Ion Trap and HPLC/ESI‐Q‐TOF MS.” Proteomics 13: 2455–2468. 10.1002/pmic.201300073. 23744580

[fsn370494-bib-0025] Iqbal, J. , W. Li , M. Hasan , et al. 2014. “Distortion of Homeostatic Signaling Proteins by Simulated Microgravity in Rat Hypothalamus: A16O/18O‐Labeled Comparative Integrated Proteomic Approach.” Proteomics 14: 262–273. 10.1002/pmic.201300337. 24323493

[fsn370494-bib-0027] Jabeen, S. , M. F. Ali , A. M. u. Din , et al. 2023. “Phytochemical Screening and Allelopathic Potential of Phytoextracts of Three Invasive Grass Species.” Scientific Reports 13, no. 1: 8080. 10.1038/s41598-023-35253-x. 37202455 PMC10195856

[fsn370494-bib-0028] Jia, Y. , Y. Ma , G. Cheng , Y. Zhang , and S. Cai . 2019. “Comparative Study of Dietary Flavonoids With Different Structures as α‐Glucosidase Inhibitors and Insulin Sensitizers.” Journal of Agricultural and Food Chemistry 67, no. 37: 10521–10533. 10.1021/acs.jafc.9b04943. 31461284

[fsn370494-bib-0029] Johnson, J. , J. Mani , N. Ashwath , and M. Naiker . 2020. “Potential for Fourier Transform Infrared (FTIR) Spectroscopy Toward Predicting Antioxidant and Phenolic Contents in Powdered Plant Matrices.” Spectrochimica Acta ‐ Part A: Molecular and Biomolecular Spectroscopy 233: 118228. 10.1016/j.saa.2020.118228. 32155578

[fsn370494-bib-0030] Khattak, S. , and H. Khan . 2016. “Phyto‐Glycosides as Therapeutic Target in the Treatment of Diabetes.” Mini‐Reviews in Medicinal Chemistry 18, no. 3: 208–215. 10.2174/1389557516666160909112751. 27629995

[fsn370494-bib-0031] Laskowski, R. A. , and M. B. Swindells . 2011. “LigPlot+: Multiple Ligand‐Protein Interaction Diagrams for Drug Discovery.” Journal of Chemical Information and Modeling 51, no. 10: 2778–2786. 10.1021/ci200227u. 21919503

[fsn370494-bib-0032] Li, W. , Z. Chen , M. Yan , P. He , Z. Chen , and H. Dai . 2016. “The Protective Role of Isorhamnetin on Human Brain Microvascular Endothelial Cells From Cytotoxicity Induced by Methylglyoxal and Oxygen‐Glucose Deprivation.” Journal of Neurochemistry 136, no. 3: 651–659. 10.1111/jnc.13436. 26578299

[fsn370494-bib-0033] Lim, L. L. , E. Chow , and J. C. N. Chan . 2023. “Cardiorenal Diseases in Type 2 Diabetes Mellitus: Clinical Trials and Real‐World Practice.” Nature Reviews Endocrinology 19, no. 3: 151–163. 10.1038/s41574-022-00776-2 36446898

[fsn370494-bib-0034] Lim, S. , and K. S. Park . 2013. “The Use of *Ginkgo Biloba* Extract in Cardiovascular Protection in Patients With Diabetes.” In Diabetes: Oxidative Stress and Dietary Antioxidants, 165–172. Academic Press.

[fsn370494-bib-0035] Lipinski, C. A. , F. Lombardo , B. W. Dominy , and P. J. Feeney . 2012. “Experimental and Computational Approaches to Estimate Solubility and Permeability in Drug Discovery and Development Settings.” Advanced Drug Delivery Reviews 64: 4–17. 10.1016/s0169-409x(00)00129-0 11259830

[fsn370494-bib-0036] Lok, K. H. , N. J. Wareham , R. S. Nair , C. W. How , and L. H. Chuah . 2022. “Revisiting the Concept of Incretin and Enteroendocrine L‐Cells as Type 2 Diabetes Mellitus Treatment.” Pharmacological Research 180: 106237. 35487405 10.1016/j.phrs.2022.106237 PMC7614293

[fsn370494-bib-0037] Maheshu, V. , D. T. Priyadarsini , and J. M. Sasikumar . 2014. “Antioxidant Capacity and Amino Acid Analysis of Caralluma Adscendens (Roxb.) Haw Var. Fimbriata (Wall.) Grav. & Mayur. Aerial Parts.” Journal of Food Science and Technology 51, no. 10: 2415–2424. 10.1007/s13197-012-0761-5. 25328180 PMC4190237

[fsn370494-bib-0038] Manzoor, Y. , M. Hasan , A. Zafar , et al. 2022. “Incubating Green Synthesized Iron Oxide Nanorods for Proteomics‐Derived Motif Exploration: A Fusion to Deep Learning Oncogenesis.” ACS Omega 7: 47996–48006. 10.1021/acsomega.2c05948. 36591177 PMC9798745

[fsn370494-bib-0039] Marahatha, R. , K. Gyawali , K. Sharma , et al. 2021. “Pharmacologic Activities of Phytosteroids in Inflammatory Diseases: Mechanism of Action and Therapeutic Potentials.” Phytotherapy Research 35, no. 9: 5103–5124. 33957012 10.1002/ptr.7138

[fsn370494-bib-0042] Mustafa, G. , and S. Komatsu . 2017. “Nanoparticles Mediated Soybean Response Mechanism at Morphological, Physiological, and Proteomic Level.” Current Proteomics 14, no. 1: 3–12. 10.2174/1570164613666161128145103.

[fsn370494-bib-0043] Mustafa, G. , and S. Komatsu . 2021. “Plant Proteomic Research for Improvement of Food Crops Under Stresses: A Review.” Molecular Omics 17, no. 6: 860–880. 34870299 10.1039/d1mo00151e

[fsn370494-bib-0041] Mustafa, G. , M. Hasan , H. Yamaguchi , K. Hitachi , K. Tsuchida , and S. Komatsu . 2020. “A Comparative Proteomic Analysis of Engineered and Bio Synthesized Silver Nanoparticles on Soybean Seedlings.” Journal of Proteomics 224: 103833. 10.1016/j.jprot.2020.103833. 32450145

[fsn370494-bib-0040] Mustafa, G. , S. K. Chaudhari , M. Manzoor , S. Batool , M. Hatami , and M. Hasan . 2024. “Zinc Oxide Nanoparticles Mediated Salinity Stress Mitigation in *Pisum Sativum* : A Physio‐Biochemical Perspective.” BMC Plant Biology 24, no. 1: 835. 10.1186/s12870-024-05554-y. 39243061 PMC11378595

[fsn370494-bib-0044] Nagarajan, D. , and R. Kumar . 2017. “Studies Fourier Transform Infrared Spectroscopy Analysis of Garlic (Allium).” Revista Internacional de Estudios de Zoología 2: 2455–7269.

[fsn370494-bib-0045] Nazir, I. , N. U. Rahman , Z. Alvi , et al. 2017. “Antidiabetic Activities of an LC/MS Fingerprinted Aqueous Extract of Fagonia Cretica Lin Preclinical Models.” Planta Medica 83, no. 14–15: 1141–1148. 10.1055/s-0043-107616. 28388787

[fsn370494-bib-0046] Newman, D. J. , and G. M. Cragg . 2020. “Natural Products as Sources of New Drugs Over the Nearly Four Decades From 01/1981 to 09/2019.” Journal of Natural Products 83, no. 3: 770–803. 32162523 10.1021/acs.jnatprod.9b01285

[fsn370494-bib-0047] Nishina, A. , D. Sato , J. Yamamoto , K. Kobayashi‐Hattori , Y. Hirai , and H. Kimura . 2019. “Antidiabetic‐Like Effects of Naringenin‐7‐O‐Glucoside From Edible Chrysanthemum ‘Kotobuki’ and Naringenin by Activation of the PI3K/Akt Pathway and PPARγ.” Chemistry and Biodiversity 16, no. 1: e1800434. 10.1002/cbdv.201800434. 30462381

[fsn370494-bib-0048] Ntie‐Kang, F. , L. L. Lifongo , J. A. Mbah , et al. 2013. “In Silico Drug Metabolism and Pharmacokinetic Profiles of Natural Products From Medicinal Plants in The Congo Basin.” In Silico Pharmacology 1, no. 1: e12. 10.1186/2193-9616-1-12. PMC4230438 25505657

[fsn370494-bib-0049] O'Boyle, N. M. , M. Banck , C. A. James , C. Morley , T. Vandermeersch , and G. R. Hutchison . 2011. “Open Babel: An Open Chemical Toolbox.” Journal of Cheminformatics 3, no. 10: 33. 10.1186/1758-2946-3-33. 21982300 PMC3198950

[fsn370494-bib-0050] Ozturk, M. , V. Altay , A. Latiff , et al. 2018. “A Comparative Analysis of the Medicinal Plants Used for Diabetes Mellitus in the Traditional Medicine in Turkey, Pakistan, and Malaysia.” Plant and Human Health 1: 409–461.

[fsn370494-bib-0051] Panda, S. , and A. Kar . 2007. “Antidiabetic and Antioxidative Effects of *Annona Squamosa* Leaves Are Possibly Mediated Through Quercetin‐3‐O‐Glucoside.” BioFactors 31, no. 3–4: 201–210. 10.1002/biof.5520310307. 18997283

[fsn370494-bib-0052] Pawaskar, M. , S. P. Bilir , S. Kowal , C. Gonzalez , S. Rajpathak , and G. Davies . 2019. “Cost‐Effectiveness of DPP‐4 Inhibitor and SGLT2 Inhibitor Combination Therapy for Type 2 Diabetes.” American Journal of Managed Care 25, no. 5: 231–238. 31120717

[fsn370494-bib-0053] Pettersen, E. F. , T. D. Goddard , C. C. Huang , et al. 2004. “UCSF Chimera—A Visualization System for Exploratory Research and Analysis.” Journal of Computational Chemistry 25, no. 13: 1605–1612. 15264254 10.1002/jcc.20084

[fsn370494-bib-0054] Poodineh, J. , A. Khazaei Feizabad , and A. Nakhaee . 2015. “Antioxidant Activities of Caralluma Tuberculata on Streptozotocin‐Induced Diabetic Rats.” Drug Development Research 76, no. 1: 40–47. 10.1002/ddr.21239. 25620374

[fsn370494-bib-0055] Poodineh, J. , and A. Nakhaee . 2016. “Hypoglycemic and Hypolipidemic Effects of Caralluma Tuberculata and Its Safety on Liver and Kidneys of Diabetic Rats.” Turkish Journal of Biochemistry 41, no. 3: 136–143. 10.1515/tjb-2016-0023.

[fsn370494-bib-0056] Qasim, S. , A. Zafar , M. S. Saif , et al. 2020. “Green Synthesis of Iron Oxide Nanorods Using Withania Coagulans Extract Improved Photocatalytic Degradation and Antimicrobial Activity.” Journal of Photochemistry and Photobiology, B: Biology 204: 111784. 10.1016/j.jphotobiol.2020.111784. 31954266

[fsn370494-bib-0057] Quek, A. , N. K. Kassim , P. C. Lim , et al. 2021. “α‐Amylase and Dipeptidyl Peptidase‐4 (DPP‐4) Inhibitory Effects of Melicope Latifolia Bark Extracts and Identification of Bioactive Constituents Using In Vitro and In Silico Approaches.” Pharmaceutical Biology 59, no. 1: 962–971. 10.1080/13880209.2021.1948065. PMC8344235 34347568

[fsn370494-bib-0079] Rauf, A ., W. U. Rehman , M. R. Jan , N. Muhammad . 2013. “Phytochemical, Phytotoxic and Antioxidant Profile of Caralluma Tuberculata N. E. Brown. J. Wudpecker.” Journal of Pharmacy and Pharmacology 2, no. 2: 21–25.

[fsn370494-bib-0058] Rizevsky, S. , M. Matveyenka , and D. Kurouski . 2022. “Nanoscale Structural Analysis of a Lipid‐Driven Aggregation of Insulin.” Journal of Physical Chemistry Letters 13, no. 10: 2467–2473. 10.1021/acs.jpclett.1c04012. 35266717 PMC9169669

[fsn370494-bib-0059] Sadaf, M. , G. Akram , M. Inc , M. Dawood , H. Rezazadeh , and A. Akgül . 2023. “Exact Special Solutions of Space‐Time Fractional Cahn‐Allen Equation by Beta and M‐Truncated Derivatives.” International Journal of Modern Physics B: Condensed Matter Physics, Statistical Physics, Applied Physics 38: 2450118. 10.1142/S0217979224501182.

[fsn370494-bib-0060] Saif, M. S. , A. Zafar , M. Waqas , et al. 2021. “Phyto‐Reflexive Zinc Oxide Nano‐Flowers Synthesis: An Advanced Photocatalytic Degradation and Infectious Therapy.” Journal of Materials Research and Technology 13: 2375–2391. 10.1016/j.jmrt.2021.05.107.

[fsn370494-bib-0061] Sander, T. , J. Freyss , M. Von Korff , and C. Rufener . 2015. “DataWarrior: An Open‐Source Program for Chemistry Aware Data Visualization and Analysis.” Journal of Chemical Information and Modeling 55, no. 2: 460–473. 10.1021/ci500588j. 25558886

[fsn370494-bib-0062] Shastri, M. A. , and R. V. Gadhave . 2022. “Recent Update on Pyrimidine Derivatives as Potential DPP‐Iv Inhibitors for the Treatment of Type 2 Diabetes Mellitus.” Journal of Pharmaceutical Negative Results 13: 5734–5743.

[fsn370494-bib-0063] Shehadeh, M. B. , G. A. R. Y. Suaifan , and A' M. Abu‐Odeh . 2021. “Plants Secondary Metabolites as Blood Glucose‐Lowering Molecules.” Molecules 26, no. 14: 4333. 34299610 10.3390/molecules26144333 PMC8307461

[fsn370494-bib-0065] Shehzad, J. , A. Emili , J. Kwan , et al. 2024. “Lead Toxicity Regulation via Protein Degradation and Tetrapyrrole Biosynthesis Pathways in Brassica Species: A Comparative Quantitative Analysis of Proteomic Study.” Plant Physiology and Biochemistry 213: 108867. 10.1016/j.plaphy.2024.108867. 38936069

[fsn370494-bib-0064] Shehzad, J. , I. Khan , S. Zaheer , A. Farooq , S. K. Chaudhari , and G. Mustafa . 2023. “Insights Into Heavy Metal Tolerance Mechanisms of Brassica Species: Physiological, Biochemical, and Molecular Interventions.” Environmental Science and Pollution Research 30, no. 50: 108448–108476. 37924172 10.1007/s11356-023-29979-4

[fsn370494-bib-0066] Shehzadi, S. , S. M. Khan , G. Mustafa , et al. 2022. “Antiviral COVID‐19 Protein and Molecular Docking: In Silico Characterization of Various Antiviral Compounds Extracted From Arisaema Jacquemontii Blume.” Frontiers in Public Health 10: 964741. 10.3389/fpubh.2022.964741. 36211701 PMC9540392

[fsn370494-bib-0067] Sultan, K. , M. Zakir , H. Khan , et al. 2014. “The Effect of Extract/Fractions of Caralluma Tuberculata on Blood Glucose Levels and Body Weight in Alloxan‐Induced Diabetic Rabbits.” Journal of Evidence‐Based Complementary & Alternative Medicine 19, no. 3: 195. 10.1177/2156587214531019. 24742609

[fsn370494-bib-0068] Tanwar, O. , L. Tanwar , M. Shaquiquzzaman , M. M. Alam , and M. Akhter . 2014. “Structure Based Virtual Screening of MDPI Database: Discovery of Structurally Diverse and Novel DPP‐IV Inhibitors.” Bioorganic and Medicinal Chemistry Letters 24, no. 15: 3447–3451. 10.1016/j.bmcl.2014.05.076. 24948564

[fsn370494-bib-0069] Trott, O. , and A. J. Olson . 2010. “AutoDock Vina: Improving the Speed and Accuracy of Docking With a New Scoring Function, Efficient Optimization, and Multithreading.” Journal of Computational Chemistry 31, no. 2: 455–461. 10.1002/jcc.21334. 19499576 PMC3041641

[fsn370494-bib-0070] Veber, D. F. , S. R. Johnson , H. Y. Cheng , B. R. Smith , K. W. Ward , and K. D. Kopple . 2002. “Molecular Properties That Influence the Oral Bioavailability of Drug Candidates.” Journal of Medicinal Chemistry 45, no. 12: 2615–2623. 10.1021/jm020017n. 12036371

[fsn370494-bib-0071] Wallace, A. C. , R. A. Laskowski , and J. M. Thornton . 1995. “Ligplot: A Program to Generate Schematic Diagrams of Protein‐Ligand Interactions.” Protein Engineering, Design and Selection 8, no. 2: 127–134. 10.1093/protein/8.2.127. 7630882

[fsn370494-bib-0072] Wang, G. X. , F. L. Cao , and J. Chen . 2006. “Progress in Researches on the Pharmaceutical Mechanism and Clinical Application of *Ginkgo Biloba* Extract on Various Kinds of Diseases.” Chinese Journal of Integrative Medicine 12, no. 3: 234–239. 17005090 10.1007/BF02836532

[fsn370494-bib-0073] Wu, W. L. , J. Hao , M. Domalski , et al. 2016. “Discovery of Novel Tricyclic Heterocycles as Potent and Selective DPP‐4 Inhibitors for the Treatment of Type 2 Diabetes.” ACS Medicinal Chemistry Letters 7, no. 5: 498–501. 10.1021/acsmedchemlett.6b00027. 27190600 PMC4867478

[fsn370494-bib-0074] Zafar, S. , A. Farooq , S. Batool , T. Tariq , M. Hasan , and G. Mustafa . 2024. “Green Synthesis of Iron Oxide Nanoparticles for Mitigation of Chromium Stress and Anti‐Oxidative Potential in *Triticum Aestivum* .” Hybrid Advances 5: 100156. 10.1016/j.hybadv.2024.100156.

[fsn370494-bib-0075] Zaheer, S. , J. Shehzad , S. K. Chaudhari , M. Hasan , and G. Mustafa . 2023. “Morphological and Biochemical Responses of *Vigna Radiata* L. Seedlings Towards Green Synthesized SiO2 NPs.” Silicon 15: 5925–5936. 10.1007/s12633-023-02470-y.

[fsn370494-bib-0076] Zhou, H. , W. Tang , J. Zeng , and C. Tang . 2014. “Screening of Terpene Lactones and Flavonoid Glycosides in *Gingko Biloba* Capsule by UPLC‐ Orbitrap High Resolution MS, With Emphasis on Isomer Differentiation.” Journal of Food and Nutrition Research 2, no. 7: 369–376. 10.12691/jfnr-2-7-7.

[fsn370494-bib-0077] Zito, P. , M. Sajeva , M. Bruno , et al. 2010. “Essential Oil Composition of Stems and Fruits of Caralluma Europaea N.E.Br. (Apocynaceae).” Molecules 15, no. 2: 627–638. 10.3390/molecules15020627. 20335933 PMC6256926

