# Supplementary material for: Apteranthes tuberculata's Antidiabetic Potential: Exploring Phytochemicals, Screening Antioxidant Activity, and Validating DPP‐4 Inhibition Using In Vitro and In Silico Approaches
Source: Food Sci Nutr. 2025 Jul 24;13(7):e70494. doi: 10.1002/fsn3.70494 (PMC12288619; doi:10.1002/fsn3.70494)
Supplement: Supplementary file 1 — Data S1. [file FSN3-13-e70494-s001.docx]

**Supplementary Tables**

**Suppl. Table 1:** Qualitative phytochemical screening of plant extract.

| **S.No** | **Phyto-constituents** | **Inference** |
| --- | --- | --- |
| 1 | Terpenoids | - |
| 2 | Flavonoids | +++ |
| 3 | Tannins | ++ |
| 4 | Glycosides | - |
| 5 | Alkaloids | + |
| 6 | Saponins | ++ |
| 7 | Cardiac glycoside | - |
| 8 | Steroids | - |
| 9 | Phenol | - |
| 10 | Coumarin | + |
| 11 | Emodin | - |
| 12 | Anthocyanin | - |
| 13 | Betacyanin | + |
| 14 | Protein | + |
| 15 | Fats and oils | + |
| 16 | Phytosterols | +++ |
| 17 | Phlobatannins | - |
| 18 | Anthraquinone glycosides | - |

**Key: (+):** Present, **(-):** Absent

**Suppl. Table 2:** Total phenolic and flavonoid contents of *A. tuberculata*.

| **S.No** | **Plant sample** | **Total phenolics expressed as gallic acid equivalent (mg/g)** | **Total flavonoids expressed as quercetin equivalent (mg/g)** |
| --- | --- | --- | --- |
| 1 | CTME | 71.991 ± 0.78 | 66.2162 ± 0.09 |

Result is expressed as mean ± standard deviation (n = 3)

**Key:** ATME; *Apteranthes tuberculata* methanolic extract

**Suppl. Table 3:** Two-dimensional chemical structural representation of the selected dataset.

|  Deoxyguanosine |  Isorhamnetin |  Bilobalide |
| --- | --- | --- |
|  Ginkgolide J | Kaempferol-7-*O*-glucoside  |  Quercetin-3-*O*-glucoside |
|  Caratuberside E |  Kaempferol-7-*O*-rutinoside | Quercetin-3-*O*-rutinoside  |
|  Isorhamnetin-3-*O*-rutinoside | Russelioside C  |  Kaempferol-3-O-rutinoside-7-O-glucoside |
|  Russelioside G | Digoxin  | Russelioside B  |
|  Ginkgolide C | Pregnane glycoside  | Bouceroside – ADC  |
| Russelioside F  | Caratuberside E  | Raratuberside C  |
| Isopropenyl Glycopyranosides  | Tragopogonsaponin M  |  12,20-di-*O*-benzoyl boucerin |

**Suppl. Table 4: FT-IR frequency range and functional groups in A. tuberculate**

| **Sr.#** | **Absorbance (cm^-1^)** | **Bond** | **Functional group** | **Major compound** |
| --- | --- | --- | --- | --- |
| 1 | 3323 (m) | O-H stretch | Alcohol/Phenol | Carbohydrate Amino acid |
| 2 | 2980 (m) | O-H stretch | Alcohol/Phenol | Carbohydrate Amino acid |
| 3 | 2925 (m) | O-H stretch | Alcohol/Phenol | Carbohydrate Amino acid |
| 4 | 1638 (m) | N-H bend | Amines | Protein |
| 5 | 1379 (m) | C-H bend | Alkanes | Glycogen |
| 6 | 1274 (s) | C-N stretch | Aliphatic amines | Amino acids |
| 7 | 1044 (m) | C-N stretch | Aliphatic amines | Amino acids |
| 8 | 997 (m) | =C-H bend | Alkenes | Lipids |
| 9 | 825 (m) | C-H stretch | Aromatic | Phenolic |
| 10 | 712 (m) | C-H stretch | Aromatic | Phenolic |
| 11 | 606 (m) | C-Cl stretch | Alkyl halide | Halo Alkanes |
| 12 | 591 (m) | C-Cl stretch | Alkyl halide | Chloro compound |
| 13 | 575 (m) | C-Cl stretch | Alkyl halide | Chloro compound |
| 14 | 563 (m) | C-Cl stretch | Alkyl halide | Chloro compound |
| 15 | 548 (m) | C-Br stretch | Alkyl halide | Bromo compound |
| 16 | 535 (m) | C-Br stretch | Alkyl halide | Bromo compound |
| 17 | 526 (m) | C-Br stretch | Alkyl halide | Bromo compound |
| 18 | 520 (m) | C-Br stretch | Alkyl halide | Bromo compound |

**Suppl. Table 5:** Summary of molecular docking results of the selected dataset of chemical compounds.

| **Chemical compounds** | **Binding energy** | **Hydrophobic interaction** | | **Hydrogen bonding interaction** | | | **Total number of bonds** |
| --- | --- | --- | --- | --- | --- | --- | --- |
|  |  | **Interacting residues** | **No. of bonds** | **Interactions** | **Distance** | **No of bonds** |  |
| **Deoxyguanosine** | -5.9 | Ser630(1), Tyr631(2),Tyr666(3),  Tyr662(3), Val711(1), His740(2) | 12 | O1-Tyr547: OH | 2.84 | 1 | 13 |
| **Isorhamnetin** | -8.0 | Ser630(3), Tyr631(2), Trp659(1), Val656(1), Tyr666(2), Tyr662(5), Val711(3), Tyr547(1), Phe357(4), Glu206(2), Arg125(3), Glu205(3), His740(3), Asn710(2) | 35 | O2-Asn710:ND2  O6-His740NE2  O5-Glu205:OE2 | 2.95  2.86  2.78 | 3 | 38 |
| **Bilobalide** | -7.4 | Lys71(1), Asn92(4), Asp96(1), Ser101(1),Ile102(5),Asn74(4), Phe95(3), Leu90(1) | 20 | O7-Asn92:O  O5-Ser101: OG | 2.92  3.21 | 2 | 22 |
| **Ginkgolide J** | -8.7 | Ser101(1), Asn92(2), Phe95(5), Ile102(6),Leu90(2),Ile76(1), Lys71(3),Asn74(8), | 28 | O4-Asn74:ND2  O9-Asn74:O  O6-Lys71:NZ  O9-Lys71:NZ | 3.18  3.00  2.90  2.80 | 4 | 32 |
| **Kaempferol-7-*O*-glucoside** | -8.2 | Val207(2), Glu206(3),Glu205(4), Arg125(1),His740(1),Ser630(4), Trp629(3),Tyr547(3),Phe357(4), Ser209(3) | 28 | O5-Val207:O  O9-Glu206:OE1  O9-Glu205:OE2  O11-His740:NE2  O11-Ser630: OG  O5-Ser209: OG  O6-Ser209: OG | 2.98  2.89  2.82  2.96  2.89  2.97  2.91 | 7 | 35 |
| **Quercetin-3-*O*-glucoside** | -7.8 | Phe357(4), Glu206(5),Arg125(1), His740(1),Ser630(1),Tyr662(1), Tyr547(6),Cys551(1),Tyr585(2), Glu205(5) | 27 | O9-Glu205:C  O10-Glu205:C  O5-Glu205:OE2  O9-Ser209: OG  O10-Glu206:OE1  O5-Glu206:OE2  O7-His740:NE2  O7-Ser630: OG  O6-Tyr662: OH  O5-Tyr662: OH  O12-Tyr585: OH | 3.23  2.9  2.79  3.02  2.86  3.04  3.16  3.04  3.18  2.82  2.97 | 11 | 38 |
| **Caratuberside E** | -8.7 | Asp737(6), Val121(1),Lys122(5), Lys250(1),Gln153(5),Asn169(2), Glu191(5),Thr129(1),Gln123(3), Tyr238(1),Trp124(2),Ala707(3), Asp709(1),Asp739(3),Phe240(3) | 42 | O14-Gln153:NE2  O15-Gln153:NE2  O14-Asn170:ND2 | 3.01  3.08  2.98 | 3 | 45 |
| **Kaempferol-7-*O*-rutinoside** | -9.4 | Asp302(3), Val303(2),Thr304(5), Ser217(1),Trp305(3),Trp157(2), Pro218(2),Pro159(4),Trp215(2), Phe208(1),Ala210(2),Trp154(1), Ser212(5),Trp216(2),Arg358(3) | 38 | O8-Asp302:OD2  O14-Asp302:OD2  O8-Val303:O  O8-Thr304:OG1  O7-Trp305: N  O7-Trp305:O  O11-Leu214:O  O12-Ser212: OG  O13-Ser212: OG  O14-Arg358:NH2  O15-Arg358:NH2  O14-Arg358:NH1 | 3.11  2.98  2.87  2.57  2.99  2.73  2.89  2.86  3.02  2.83  3.10  3.16 | 12 | 50 |
| **Quercetin-3-*O*-rutinoside** | -8.8 | Tyr48(1), Asn562(3),Tyr752(2), Val546(3),Arg125(1),Tyr547(8), Ser630(3),Gly741(1),Lys554(4), Trp629(7),Asp545(1),Trp627(3) | 37 | O12-Asn562:ND2  O11-Tyr752: OH  O4-Arg125:NH2  O2-Ser630: OG  O5-Lys554:NZ  O13-Lys554:NZ  O14-Lys554:NZ  O14-Asp545:OD1  O14-Asp545:OD2 | 3.15  3.13  3.29  3.03  2.93  3.04  3.32  3.07  3.12 | 9 | 46 |
| **Isorhamnetin-3-*O*-rutinoside** | -8.9 | Gly380(4), Asp588(3),Ser349(4), Val354(2),Glu347(4),Gly355(1), Met348(3),Ile375(2),Glu378(3), Thr351(7),Thr350(4),Glu379(1), Ser376(16) | 53 | O13-Ser376: OG  O13-Asp588:OD2  O7-Ser349: OG  O8-Glu347:OE2  O8-Gly355: N  O9-Glu378:OE1  O14-Thr351:OG1 | 3.16  3.04  3.09  3.10  2.80  2.89  3.24 | 7 | 60 |
| **Russelioside C** | -8.9 | Tyr662(3), Glu205(3),Glu206(1), Ser209(2),Arg356(1),Glu361(6), Ser360(2),Ile374(3),Ile405(2), Phe357(8),Tyr547(1),Arg358(2), Tyr666(1),Arg125(4),Ser630(1), His740(1) | 41 | O11-Tyr662: OH  O12-Tyr662: OH  O12-Glu205:OE2  O12-Glu206:OE2  O5-Ser209: OG  O4-Ile405:O  O10-Ser630: OG  O10-His740:NE2 | 2.77  3.14  2.87  3.01  3.02  2.70  2.79  3.11 | 8 | 49 |
| **Kaempferol-3-*O*-rutinoside-7-*O*-glucoside** | -9.4 | Asp302(3), Val303(2),Thr304(5), Ser217(1),Trp305(3),Trp157(2), Pro218(2),Pro159(4),Trp215(2), Phe208(1),Ala210(2),Trp154(1), Ser212(5), Trp216(2), Arg358(3) | 38 | O8-Asp302:OD2  O14-Asp302:OD2  O8-Val303:O  O8-Thr304:OG1  O7-Trp305: N  O7-Trp305:O  O11-Leu214:O  O12-Ser212: OG  O13-Ser212: OG  O14-Arg358:NH2  O15-Arg358:NH2  O14-Arg358:NH1 | 3.11  2.98  2.87  2.57  2.99  2.73  2.89  2.86  3.02  2.83  3.10  3.16 | 12 | 50 |
| **Russelioside G** | -10.3 | Leu214(1), Trp215(2),Trp216(5), Trp305(3),Thr304(1),Phe364(1), His363(1),Leu410(4),Ala465(2), Ala409(2),Glu464(1),Lys463(3), Phe461(1),Pro159(3),Trp157(2), Thr156(4) | 36 | O3-Trp216:O  O12-Leu410:O  O14-Ala465: N  O14-Ser462:O  O14-Lys463:O  O13-Lys463:O  O2-Thr156:OG1 | 2.84  2.84  3.03  3.15  3.16  2.89  3.05 | 7 | 43 |
| **Digoxin** | -8.9 | Trp563(2), Tyr752(2),Trp627(1), Lys554(2),Tyr547(6),Ser209(3), Arg358(6),Trp629(1),Phe357(5), Asn562(5),Tyr48(1),Ala564(1) | 35 | O4-Trp563: N  O11-Ser209: OG  O12-Ser209: OG  O14-Arg358:NE2  O6-Ala564: N | 3.11  3.01  2.94  2.98  3.18 | 5 | 40 |
| **Russelioside B** | -9.4 | **Very complex to draw interaction plots** | | | | | |
| **Ginkgolide C** | -8.8 | Glu738(2), Asp739(4),Trp124(1), Lys122(4),Gln123(2),Asp709(1), Phe240(7), Asp737(1) | 22 | O10-Asp739:OD2  O10-Lys122:NZ  O9-Gln123:O | 3.17  2.84  2.77 | 3 | 25 |
| **Pregnane glycoside** | -10.8 | **Very complex to draw interaction plots** | | | | | |
| **Bouceroside–ADC** | -9.8 | Thr365(1), Trp305(3),Thr304(2), Leu214(1),Trp216(7),Pro159(2), Arg61(3),Ser106(5),Trp157(3), Thr156(1),Leu366(1),Leu410(1), Ala306(4), Phe364(3) | 37 | O3-Thr365:O  O13-Trp216: N  O11-Ser106: OG  O12-Ser106: OG  O15-Ser106: OG  O8-Thr156:OG1  O3-Ala306:O | 3.07  3.30  2.73  3.13  2.7  3.01  2.98 | 7 | 44 |
| **Russelioside F** | -10.0 | Thr304(3), Trp215(1),Leu214(1), Thr156(1),Trp216(1),Pro159(1), Trp157(5),Pro109(1),Arg61(1), Ser106(2),Trp305(2),His363(3), Phe364(3), Leu410(5) | 30 | O8-Thr156:OG1  O13-Ile107:O  O14-Ile107:O  O14-Ser106: OG | 2.99  3.27  3.08  3.22 | 4 | 34 |
| **Caratuberside E** | -8.3 | Ser360(1), Glu361(1),Arg358(3), Ile374(2),Gly406(1),Phe357(4), Glu408(5),Phe461(4),Ser462(1), Lys463(3),Trp62(1),Val459(1), Ile405(6), Ser209(2), Arg356(5) | 40 | O17-Lys463:O  O3-Ile405:O | 2.96  2.73 | 2 | 42 |
| **Raratuberside C** | -12.6 | **Very complex to draw interaction plots** | | | | | |
| **Isopropenyl Glycopyranosides** | -7.2 | **Very complex to draw interaction plots** | | | | | |
| **Tragopogonsaponin M** | -12 | **Very complex to draw interaction plots** | | | | | |
| **12,20-di-*O*-benzoyl boucerin** | -10.9 | Ser212(2),Trp154(3),Trp215(2), Thr156(3),Pro159(1),Phe208(3), Arg358(2),Glu361(6),Thr304(2), Trp305(2),His363(2),Pro218(3), Trp216(3) | 34 | O7-Ser212: OG  O2-Thr156:OG1  O12-Pro362:O  O8-Trp305:O | 2.70  2.96  2.82  2.97 | 4 | 38 |
